# The direct and indirect pathways of the basal ganglia antagonistically influence cortical activity and perceptual decisions

**DOI:** 10.1016/j.isci.2024.110753

**Published:** 2024-08-22

**Authors:** Enny H. van Beest, Mohammed A.O. Abdelwahab, J. Leonie Cazemier, Chrysiida Baltira, M. Cassandra Maes, Brandon D. Peri, Matthew W. Self, Ingo Willuhn, Pieter R. Roelfsema

**Affiliations:** 1 Department of Vision and Cognition, Netherlands Institute for Neuroscience (NIN), Royal Netherlands Academy of Arts and Sciences (KNAW), Amsterdam, the Netherlands; 2 Department of Cortical Structure and Function, Netherlands Institute for Neuroscience (NIN), Royal Netherlands Academy of Arts and Sciences (KNAW), Amsterdam, the Netherlands; 3 Department of Neuromodulation and Behavior, Netherlands Institute for Neuroscience (NIN), Royal Netherlands Academy of Arts and Sciences (KNAW), Amsterdam, the Netherlands; 4 Department of Psychiatry, Amsterdam UMC, University of Amsterdam, Amsterdam, the Netherlands; 5 Department of Neurosurgery, Amsterdam UMC, University of Amsterdam, Amsterdam, the Netherlands; 6 Department of Integrative Neurophysiology, Center for Neurogenomics and Cognitive Research (CNCR), VU University, Amsterdam, the Netherlands; 7 Laboratory of Visual Brain Therapy, Sorbonne Université, Institut National de la Santé et de la Recherche Médicale, Centre National de la Recherche Scientifique, Institut de la Vision, Paris, France

**Keywords:** Molecular biology, Neuroscience, Molecular neuroscience, Cognitive neuroscience

## Abstract

The striatum, the main input nucleus of the basal ganglia, receives topographically organized input from the cortex and gives rise to the direct and indirect output pathways, which have antagonistic effects on basal ganglia output directed to the cortex. We optogenetically stimulated the direct and indirect pathways in a visual and a working memory task in mice that responded by licking. Unilateral direct pathway stimulation increased the probability of lick responses toward the contralateral, non-stimulated side and increased cortical activity globally. In contrast, indirect pathway stimulation increased the probability of responses toward the stimulated side and decreased activity in the stimulated hemisphere. Moreover, direct pathway stimulation enhanced the neural representation of a contralateral visual stimulus during the delay of the working memory task, whereas indirect pathway stimulation had the opposite effect. Our results demonstrate how these two pathways influence perceptual decisions and working memory and modify activity in the dorsal cortex.

## Highlights

• The direct and indirect pathways have antagonistic effects on perceptual decisions
• Direct pathway stimulation increases neuronal activity in the dorsal cortex
• The striatum influences the cortical representations of working memories

## Graphical abstract

**Figure:**
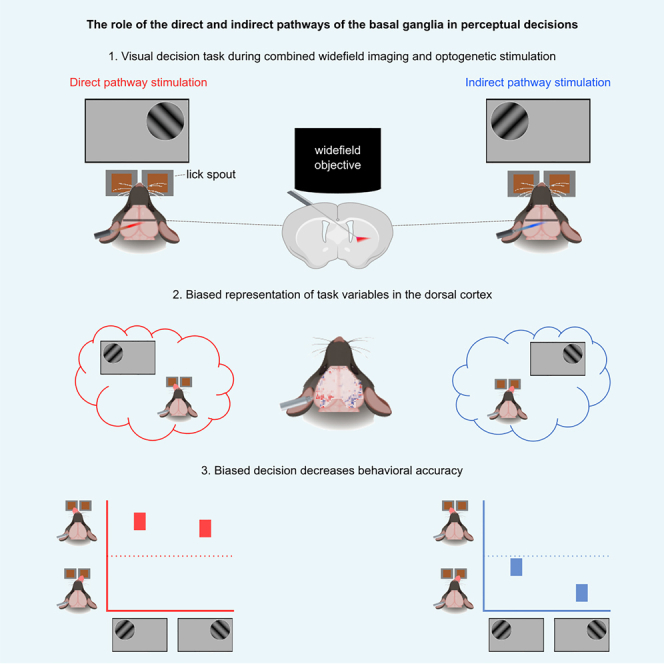

## Introduction

We constantly make decisions based on sensory information. Sometimes a stimulus requires an immediate response. In other situations, the response has to be postponed to a later point in time, so that information must be kept “online” in working memory. Previous studies in non-human primates have demonstrated that working memory is associated with delay activity in multiple brain regions, reflecting the properties of previously presented sensory stimuli.^1^^,^^2^^,^^3^^,^^4^^,^^5^^,^^6^^,^^7^^,^^8^ These results have been replicated in the mouse, where visual, multisensory, and frontal brain regions maintain activity related to previously presented stimuli.^9^^,^^10^ Importantly, persistent activity not only reflects the memory of previous stimuli but also plays a role in the planning of future behavior, decision making, and in the retrieval of associations between related concepts.^11^^,^^12^^,^^13^^,^^14^^,^^15^^,^^16^

The finding that persistent activity occurs in many brain regions suggests that it may be an emergent property of distributed brain networks.^11^^,^^17^^,^^18^ Interestingly, brain regions that are of importance in tasks that rely on working memory are often also involved in simpler decision-making tasks (e.g., tasks that do not require a delay). Example brain regions in mice involved in working memory and simple decisions include region ALM of the frontal cortex,^9^^,^^19^^,^^20^ the dorsomedial striatum,^21^^,^^22^^,^^23^ and the lateral striatum.^24^ However, it remains unclear how different brain regions orchestrate their activity to maintain working memory. The present study examines the possible role of the basal ganglia as a coordinator of persistent activity for working memory.

The cortex, basal ganglia, and thalamus are organized in an anatomical loop. Different cortical regions project to distinct regions of the striatum,^25^ which is the primary input structure of the basal ganglia. This relative segregation of cortical input streams is maintained throughout successive basal ganglia nuclei,^24^^,^^26^^,^^27^^,^^28^^,^^29^ which ultimately project to the thalamus where information is looped back to the cortical regions that provided the initial input.^30^ In line with this connectivity pattern, activity in different striatal domains correlates with activity in specific regions of the cortex.^31^^,^^32^ The activity of neurons in dorsomedial regions of the striatum correlates with activity in visual areas and midline frontal regions, whereas the activity of neurons in dorsolateral regions of the striatum correlate with activity in motor regions such as the anterior lateral motor cortex (ALM).

Previous studies have suggested that these loops from the cortex through the basal ganglia could play a role in the maintenance of persistent activity for working memory.^22^^,^^33^ Consistent with this view, the nuclei of the basal ganglia are connected with frontal cortical regions that exhibit persistent activity during working memory tasks,^34^ and striatum-projecting neurons in the medial prefrontal cortex contribute to the maintenance of spatial working memory.^35^ Furthermore, neurons of the basal ganglia themselves exhibit persistent activity during working memory tasks.^36^^,^^37^^,^^38^

**Figure 1:**
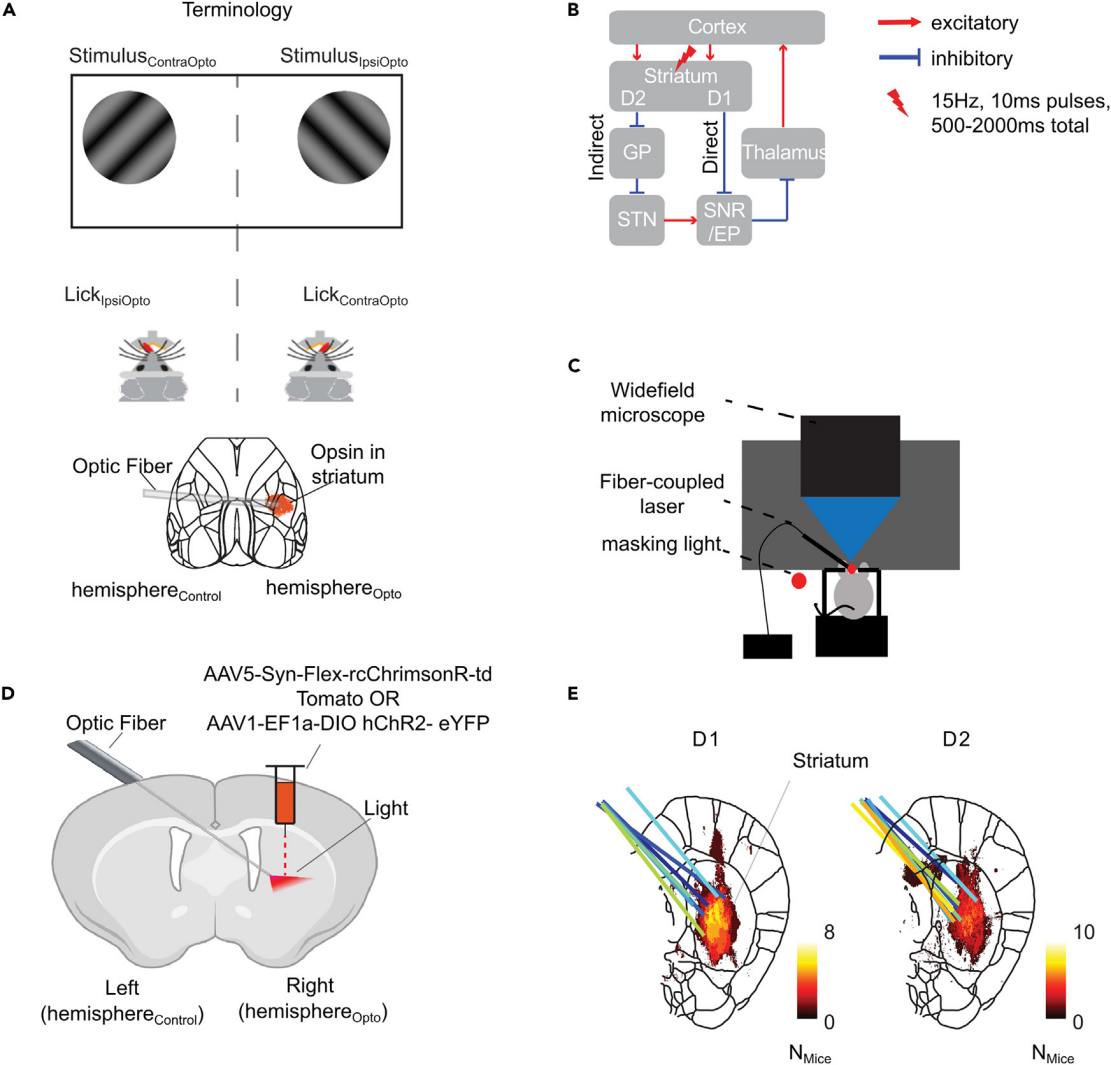
Optogenetic stimulation of the striatum during widefield imaging of the cortex (A) We optogenetically stimulated the striatum of right hemisphere (hemisphere_Opto_). The view of the cortex of the non-stimulated hemisphere_Control_ was partly occluded by the ferrule with the optic fiber and the cement to hold it in place. Visual stimuli were presented in the hemifield contralateral (stimulus_ContraOpto_) or ipsilateral (stimulus_IpsiOpto_) to hemisphere_Opto_. During behavioral tasks, mice could lick a spout in a contraversive or ipsiversive direction relative to hemisphere_Opto_. (B) Schematic of the loop from cortex to basal ganglia, then to the thalamus and back to cortex. We expressed an excitatory opsin in the direct (dSPN neurons in D1-cre mice) or indirect (iSPN neurons in D2-cre mice) pathway. (C) Set-up for widefield calcium imaging during optogenetic stimulation. Mice were head-fixed under the widefield microscope and a fiber coupled to a laser was aimed at the striatum. A masking light of approximately the same wavelength blinked at the same frequency and with the same pulse width as the laser, but at random time points. (D) A viral vector was injected in the striatum of hemisphere_Opto_ of D1-cre or D2-cre mice. The tip of the optic fiber ended above the region with virus expression (image created with BioRender). (E) Expression of the excitatory opsin in the striatum (coronal slice +0.14mm anterior to Bregma) of D1-cre (left) and D2-cre (right) mice. Histology from individual mice was aligned to the Allen Brain atlas. The color depicts the number of mice for which a certain pixel showed expression of the excitatory opsin above a threshold (see STAR Methods). The colored lines show the tracts of the optic fiber in individual mice (for expression in individual mice see Figure S1).

The cerebral cortex projects to GABAergic neurons in the striatum that belong to the antagonistic direct and indirect pathways (Figure 1B), which express different dopamine receptors. Direct pathway striatal projection neurons (dSPNs) primarily express D1-dopamine receptors, whereas indirect pathway projection neurons (iSPNs) primarily express D2-dopamine receptors^39^ (see Table S1 for abbreviations). The dSPNs and iSPNs have opposite effects on activity in basal ganglia output structures, leading to different influences on action initiation and execution.^40^^,^^41^ In rodents, dSPNs inhibit neurons in the substantia nigra pars reticulata and the entopeduncular nucleus^42^ (Figure 1B). These structures, in turn, inhibit nuclei of the brainstem, the superior colliculus and the thalamus.^22^ Hence, the direct pathway has two inhibitory synapses so that dSPNs have an overall disinhibitory effect on neurons in the brainstem, the superior colliculus, and the thalamus. In contrast, the iSPNs inhibit neurons in the globus pallidus, which inhibits the substantia nigra pars reticulata and entopeduncular nucleus.^42^ Hence, there is a third GABAergic connection in the indirect pathway, and the net effect of iSPNs on the brainstem, the superior colliculus, and the thalamus is inhibitory,^24^^,^^43^ which can explain their possible role in the inhibition of actions.^44^

Besides receiving input from all cortical areas, the striatum also receives strong dopaminergic inputs, which influence synaptic plasticity and allow the basal ganglia circuits to adapt during reinforcement learning.^45^^,^^46^^,^^47^ Indeed, the inputs from the cortex to the striatum are potentiated when new tasks are learned.^31^^,^^48^^,^^49^

The position and organization of the basal ganglia enables them to coordinate decision making. They receive sensory input from the cortex, reward and motivation signals through dopaminergic inputs, and they can gate whether activity is fed back to the cortex via the thalamocortical loop by balancing inputs via the direct and indirect pathways. Furthermore, computational studies suggested that the basal ganglia could control the contents of working memory by switching the two complementary pathways on and off.^50^^,^^51^ It is therefore of great interest to better understand the role of the basal ganglia in perceptual decisions, working memory, and its influence on cortical activity.

In this study, we asked how the basal ganglia influence persistent activity in the cortex related to perceptual decisions and working memory. We unilaterally expressed an excitatory opsin in either the direct or indirect pathway and used optogenetics to assess the influence of these pathways on behavioral responses and neuronal activity across the cortex with widefield imaging. We examined the role of this circuitry in various behaviors, including a visual stimulus detection task and a memory task with a delay.

## Results

We set out to identify the roles of the direct and indirect pathways in perceptual decisions and working memory. In the first experiment, we tested the influence of direct and indirect pathway activation on activity of the cerebral cortex outside a task. In the subsequent experiment, we investigated how activity of these pathways influences behavioral responses and cortical activity in a visual detection task. The last experiment examined persistent activity in the cortex during a delayed response task, and how it is influenced by the direct and indirect pathways.

We included 6 Thy1-5.17 GCaMP6f mice,^52^ 8 D1-cre, 10 D2-cre, 9 Thy1-5.17 GCaMP6f X D1-cre, and 7 Thy1-5.17 GCaMP6f X D2-cre of both sexes, aged 2–6 months at the start of the experiment (Table S2). GCaMP6f is a calcium sensor for widefield imaging, which in Thy1 mice is globally expressed in excitatory neurons.^52^^,^^53^ We measured changes in GCaMP6f fluorescence from baseline activity (ΔF/F) through the skull (clear-skull technique).^54^

To activate the direct or indirect pathway, we injected D1-cre (x Thy1-GCaMP6f) and D2-cre (x Thy1-GCaMP6f) mice with AAV5-*syn*-Flex-rcChrimsonR-tdTomato or AAV1-EF1a-DIO-hChR2-eYFP in the ventromedial part of the dorsal striatum of the right hemisphere (hemisphere_Opto_, Figure 1). Our injections targeted the region of the striatum with the highest density projections from ALM^25^ (Figure S1), because ALM contributes to tasks similar to those used here.^9^^,^^19^^,^^20^ The virus induced the expression of excitatory opsins ChrimsonR or ChR2. These proteins cause depolarization of neurons upon stimulation with light. The expression of the opsin was conditional on Cre activity, so that it occurred only in dSPNs in D1-cre mice and in iSPNs in D2-cre mice. We optogenetically stimulated the striatum at 15Hz with 10ms light pulses. We pooled the results across the two opsins, ChR2 (this opsin was not combined with imaging) and ChrimsonR, as they were similar. To direct light to the striatum of hemisphere_Opto_, we implanted an optic fiber at an angle, entering the skull in the non-injected hemisphere (hemisphere_Control_). This allowed us to image the entire dorsal cortex of hemisphere_Opto_, whereas the view of a central region of hemisphere_Control_ was partially obstructed by the implant (Figures 1A–1D). Upon completion of the experiments, we examined the expression of the opsin and the location of the optic-fiber tips (Figures 1E and S1). We only categorized D1-cre- and D2-cre-mice as such for which the opsin was expressed in the striatum and the fiber was correctly positioned to illuminate this brain region. Control mice had the same genetic background, underwent the same surgery and experimental procedures, but histology revealed no virus expression in or near the striatum (Table S2 provides details on the mice).

### The influence of direct and indirect pathway stimulation on cortical activity

**Figure 2:**
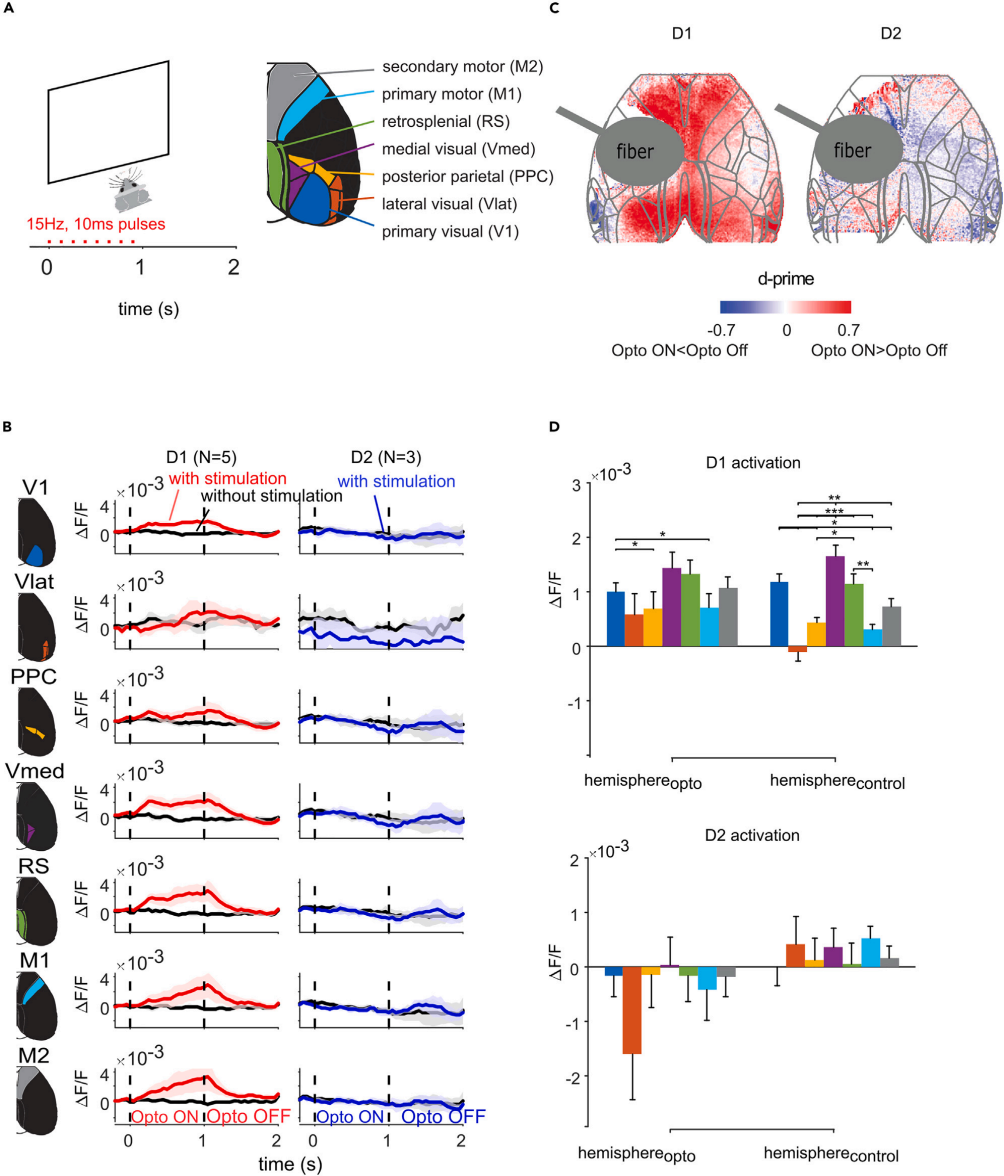
Effect of direct and indirect pathway stimulation on cortical activity (A) Left, Schematic overview of optogenetic protocol. Red line, optogenetic stimulus. Right, regions of interest. (B) Average time-course of the GCaMP signal in the areas depicted in (A) for D1-cre (left panels) and D2-cre mice (right panels). Black traces show ongoing activity and red/blue traces the activity induced by optogenetic stimulation. Shaded regions denote s.e.m. (C) The effect of stimulation was quantified with d-prime in D1-cre (left panel) and D2-cre (right panel) mice. Red colors indicate an increase, and blue colors a decrease in activity (relative to baseline). (D) ΔF/F ± s.e.m in the various areas in D1-cre (upper panel) and D2-cre (lower panel) mice during optogenetic stimulation (colors indicated as in A). Mixed-effects model with factors cortical area and hemisphere revealed a significant increase in activity for D1-cre mice (intercept: F_1,5536_ = 10.3, *p* < 0.01) and a significant main effect of area (F_6,5536_ = 2.26, *p* < 0.05). ∗,*p* < 0.05; ∗∗,*p* < 0.01; ∗∗∗,*p* < 0.001 indicate significant differences in responses to optogenetic stimulation between areas. No significant effects of optogenetic stimulation were found in D2-cre mice.

To examine how the direct and indirect pathway modulate cortical activity outside the context of a task, we optogenetically stimulated the striatum for 1 s. The mice were head-fixed and habituated to the setup, but they did not perform a task and did not have access to a lick spout. We imaged the activity in the dorsal cortex, i.e., the regions of the cortex that could be imaged through the cleared skull. We grouped activity of cortical pixels into seven regions of interest (ROIs): primary visual cortex (V1), lateral visual areas (Vlat), medial visual areas (Vmed), Posterior parietal cortex (PPC), retrosplenial cortex (RS), and primary (M1) and secondary (M2, contains ALM) motor cortex (Figure 2A). We measured the optogenetic stimulation effect as ΔF/F and by using the d-prime, which takes the variability across single trials into account (Methods).

In D1-cre-mice (*N* = 5), optogenetic stimulation of dSPNs caused a significant increase in activity (ΔF/F of the calcium signal) in most ROIs (Figures 2B–2D) (mixed effects model of stimulation induced change in ΔF/F with factors ROI and hemisphere, and mouse as random factor; intercept capturing the overall change in activity with optogenetic stimulation: F_1,5536_ = 10.2, *p* < 0.01). The most notable difference in the level of stimulation-induced activity between hemispheres was in Vlat, which was strongly activated in hemisphere_Opto_ but not in hemisphere_Control._ V1, Vmed, RS and M2 showed larger increases in activity than other ROIs (main effect of area: F_1,5536_ = 2.26, *p* < 0.05; post-hoc Wald tests of coefficients, *p* < 0.05, Figure 2D). In D2-cre-mice (*N* = 3) we did not observe a consistent change in ΔF/F activity upon iSPN stimulation outside the context of a task (mixed effects model, *p* > 0.05).

### Dorsal cortical activity during a visual detection task

**Figure 3:**
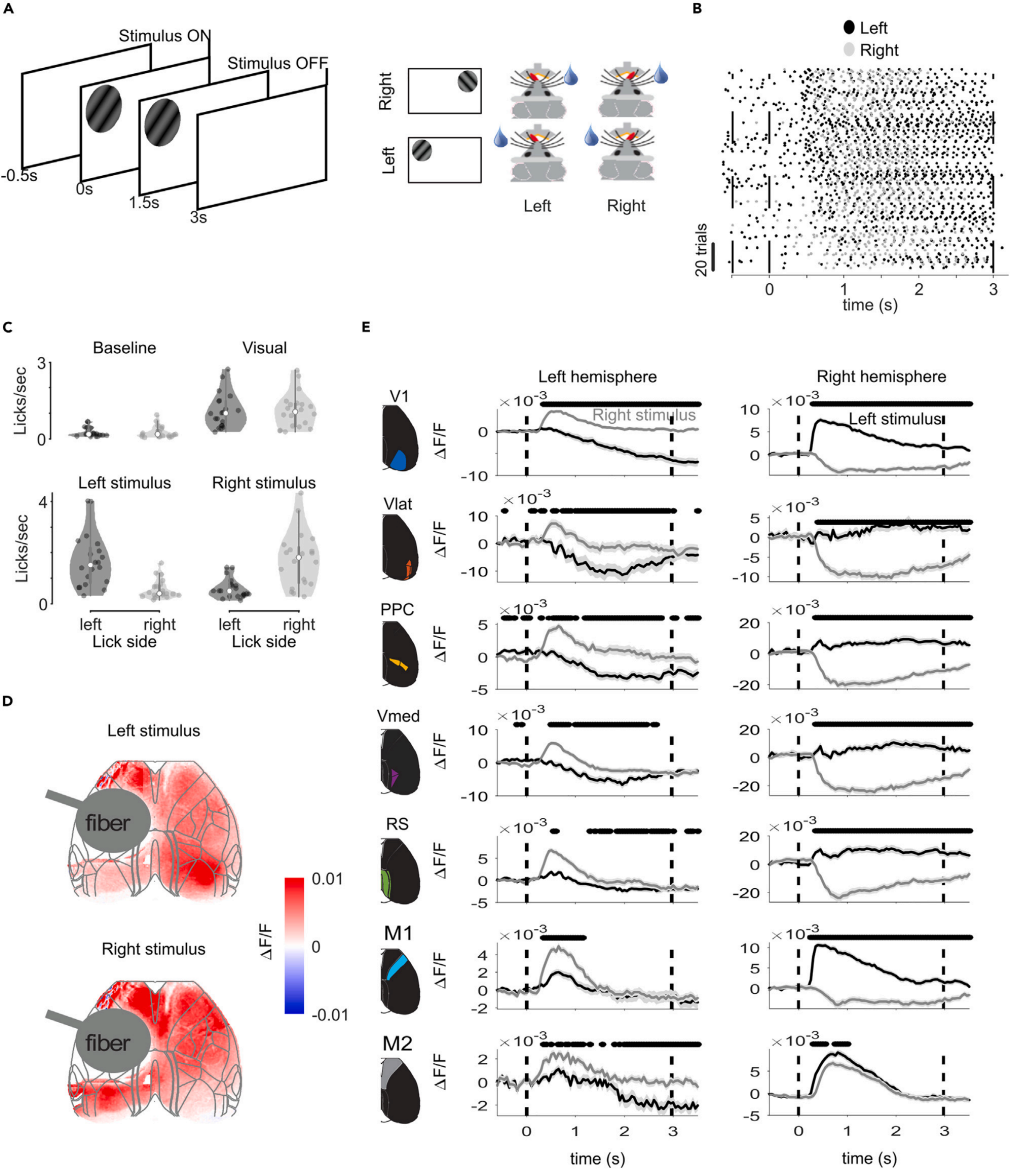
Cortical activity in the visual detection task (A) Visual detection task without optogenetic stimulation. A drifting grating appeared in the left (stimulus_ContraOpto_) or right hemifield (stimulus_IpsiOpto_) for 3s. A lick response within this time to either spout was rewarded on the same side as the visual stimulus. (B) Individual licks of an example mouse relative to stimulus onset (0s). Left licks (lick_ContraOpto_ – black) and right licks (lick_IpsiOpto_ – gray) increased over time as mice learned that the stimuli were associated with a reward. (C) Top: Lick frequency (Hz) for all mice included in this study during the baseline epoch (0-0.5s) and the first part of the visual epoch (0-1s). Mice licked more often during the visual epoch, when licks resulted in a reward. Bottom: Lick frequency in the visual epoch per visual stimulus. Mice made more leftward licks for left stimuli and vice versa. (D) Activity in the dorsal cortex of an example mouse from 0 to 1s after stimulus onset, for the left and right visual stimulus. (E) average +/− s.e.m. time courses for left (black) and right (gray) trials in different ROIs (rows) in the left and right hemisphere. Ticks above the curves indicate *p* < 0.05 in a mixed linear model per time point and area, with the side of the visual stimulus as factor.

Next, we trained eight D1-cre and eight D2-cre mice on a visual detection task. A stimulus either appeared on the left or right and the mice could lick either side of a two-sided lick spout to obtain a water reward (Figure 3A). The reward was given upon the first contact of the tongue with the spout after stimulus onset and came from the same side as the visual stimulus, even when the first lick was on the other side. Mice quickly learned to lick one of the spouts upon presentation of a visual stimulus (Figures 3B, 3C, and S2A), and to then switch to the correct side to collect the reward (Figures 3C and S2C). We first examined the pattern of cortical activity without optogenetic stimulation. As expected, the cortical regions exhibited stronger responses to contralateral stimuli (mixed effects model per ROI with visual stimulus as factor and mouse as random factor, Figures 3D and 3E), and this effect was particularly pronounced in the visual cortex.

### The direct and indirect pathways influence lick responses in a visual detection task

In 20% of the trials of the visual detection task, optogenetic stimulation started 0.5s prior to visual stimulus onset and lasted for 2 s (see Materials and Methods) and in another 20% of trials it started immediately after the first lick. The other 60% of trials were without optogenetic stimulation. In our analysis of the optogenetic effects, we will refer to a visual stimulus appearing in the hemifield contralateral to the stimulated striatum (left hemifield) as a stimulus_ContraOpto_ and to one on the same side as the stimulated striatum (right hemifield) as a stimulus_IpsiOpto_.

**Figure 4:**
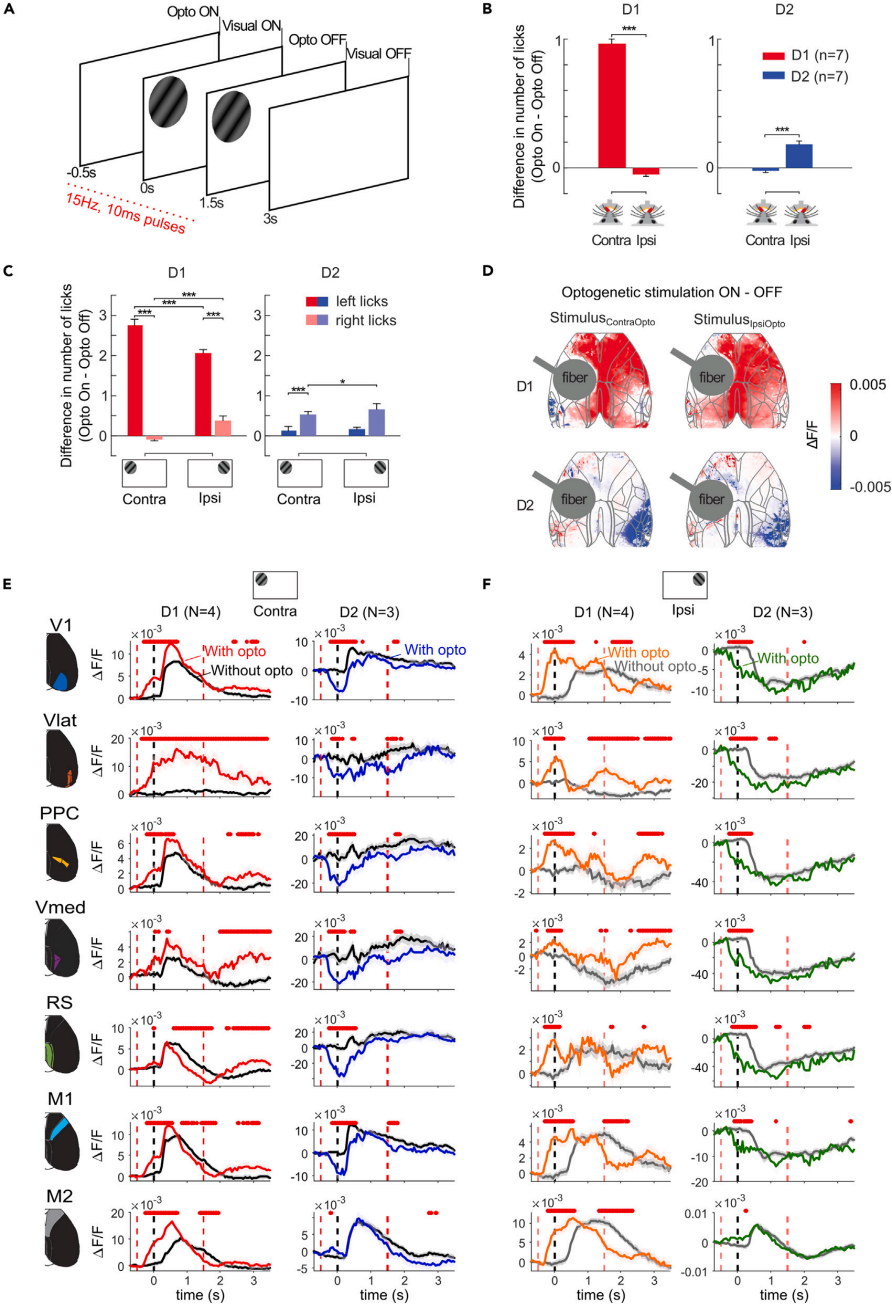
Effect of optogenetic stimulation of the striatum in the visual detection task (A) Visual detection task. A drifting grating was presented in the hemifield contralateral (stimulus_ContraOpto_) or ipsilateral (stimulus_IpsiOpto_) to the stimulated striatum (hemisphere_Opto_). Optogenetic stimulation at 15Hz (10ms pulses) occurred on 40% of the trials and started in the baseline period (i.e., −0.5 to 0s relative to the visual stimulus onset) on half of these trials, and immediately after the first lick on the other half. (B) Influence of optogenetic stimulation on the mean number of licks in the baseline period for D1-cre (red) and D2-cre (blue) mice. Error bars denote s.e.m. of the optogenetic stimulation effect. (C) Same as B, but during the presentation of stimulus_ContraOpto_ and stimulus_IpsiOpto_. Bars with darker (lighter) shade represent contraversive (ipsiversive) licks. Error bars denote s.e.m. of the difference in lick number between optogenetic stimulation on and off. (D) Average effect of optogenetic stimulation on cortical activity (time window 0-1.5s) during stimulus_ContraOpto_ (left column) and stimulus_IpsiOpto_ (right column). (E) Time-courses of GCaMP signal in ROIs of hemisphere_Opto_ in stimulus_ContraOpto_ trials in D1-cre (*N* = 4) and D2-cre mice (*N* = 3), with (red/blue) and without (black) optogenetic stimulation. The different epochs (optogenetic stimulation on, visual stimulus on, optogenetic stimulation off) are indicated by vertical dashed lines. Shaded area denotes s.e.m. Red dots, main effect of optogenetic stimulation (*p* < 0.05; mixed effects model with visual stimulus side, optogenetic stimulation, and the interaction as factors). (F) Same as in E, but now for stimulus_IpsiOpto_ trials, for trials with (orange/green) and without (gray) optogenetic stimulation.

We first analyzed the trials in which stimulation started before stimulus onset. For statistical analysis, we used a mixed effects model predicting the number of licks assuming a Poisson distribution, with factors optogenetic stimulation and lick side, and mouse as a random factor. In D1-cre mice, optogenetic stimulation caused a modest increase in the number of contraversive licks during the pre-stimulus epoch (interaction between lick side and optogenetic stimulation: F_1,7962_ = 307, *p* < 0.001; main effect of lick side: F_1,7962_ = 92, *p* < 0.001, Figure 4B). In contrast, optogenetic stimulation in D2-cre mice before stimulus appearance led to a larger increase in the number of ipsiversive licks than contraversive licks (interaction optogenetic stimulation and lick side: F_1,6956_ = 36, *p* < 0.001; main effect of lick side: F_1,6956_ = 97, *p* < 0.001).

Does stimulation of the direct and indirect pathway simply induce a lateralized licking response irrespective of context, or does the location of the visual stimulus play a role? To address this question, we focused the analysis on the effect of optogenetic stimulation in the epoch when the stimulus had appeared. In D1-cre mice, there was an interaction between optogenetic stimulation and position of the visual stimulus (mixed effects model with stimulus location as additional factor, main effect optogenetics: F_1,5834_ = 884, *p* < 0.001; interaction with visual stimulus: F_1,5834_ = 84, *p* < 0.001, and interaction with lick side: F_1,5834_ = 114, *p* < 0.001). dSPN activation increased contraversive licks for both visual stimuli, and in case of stimulus_IpsiOpto_ there was also a small increase in the number of ipsiversive licks (post-hoc Wald tests of coefficients for the difference between stimuli, *p* < 0.001; Figure 4C). The increased likelihood of licking caused by dSPN stimulation was also evident as a smaller fraction of omission trials without a lick (Figure S2B). A similar, albeit weaker interaction between optogenetics, the stimulus and lick side occurred in D2-cre mice (main effect of optogenetics: F_1,5220_ = 5.7, *p* < 0.05; three-way interaction between optogenetic stimulation, visual stimulus, and lick side: F_1,5220_ = 14.6, *p* < 0.001). Specifically, with iSPN stimulation mice made more ipsiversive licks when an ipsilateral stimulus was presented compared to when a contralateral stimulus was presented (post-hoc Wald tests of coefficients *p* < 0.05; Figure 4C). Although initial lick responses to visual stimuli were biased in a direction consistent with the genotype (Figure S2A), the interaction of lick-direction with the stimulus position implies that optogenetic stimulation did not simply force a lateralized lick response.

To further examine the influence of task engagement on the optogenetic effect, we compared the increase in the number of licks during the visual stimulus and the pre-stimulus epoch. In D1-cre mice the increase in lick number was larger during the visual stimulus (interaction between epoch, lick side, and optogenetic stimulation: F_1,10926_ = 75, *p* < 0.001; Wald tests of coefficients ps < 0.001), but this effect did not occur in D2-cre mice.

Furthermore, optogenetic stimulation did not impair the ability of mice to switch licking the spout that delivered the water when the first lick was toward the other spout. The Switch Index (SI, Equation 5) was positive (i.e., more ipsiversive switches) when the visual stimulus and reward were ipsilateral, and negative (i.e., more contraversive switches) when the visual stimulus and reward were contralateral (repeated measures ANOVA with factors visual stimulus side, genotype and optogenetic stimulation: main effect of visual stimulus side F_1,65_ = 231, *p* < 0.001, Figure S2C). Optogenetic stimulation did not change the SI. Hence, the mice switched to the other side in spite of dSPN and iSPN activation indicating that it did not enforce specific lateralized lick responses (no main or interaction effects in the ANOVA; ps > 0.05).

Taken together, these results demonstrate that the direct and indirect pathways antagonistically influence the direction of lateralized lick-responses in a manner that depends on the visual stimulus. We next examined how the activation of dSPN and iSPN neurons influences cortical activity.

### Influence of striatal pathways on cortical activity during the visual detection task

We imaged four D1-cre mice and three D2-cre mice to examine how stimulation of the direct and indirect pathways influences cortical activity during the visual-detection task. Optogenetic stimulation of dSPNs (starting 0.5s prior to visual stimulus onset and lasting for 2 s) caused a pronounced and global increase in cortical activity, which was already present in the baseline period (Figures 4D–4F and S2D and S2E). The initial effect of dSPN stimulation was an increase in cortical activity in hemisphere_Opto_, irrespective of the location of the stimulus (Figures 4E and 4F; compare red/orange and black/gray traces; red dots above the traces show a significant main effect of optogenetic stimulation; mixed effects model per time point with ∼250 trials per mouse, ps < 0.05). After this initial increase in cortical activity, the effect of dSPN stimulation varied across ROIs of hemisphere_Opto_ (Figures 4E and 4F). In hemisphere_Control_ we observed a global suppression after the initial activity increase when a stimulus was presented ipsilateral to this hemisphere (Figure S2D). Conversely, iSPN activation prior to stimulus onset briefly decreased activity in most of hemisphere_Opto_, and briefly increased activity in several ROIs of hemisphere_Control_ (ps < 0.05 for subsequent time points) (Figures 4E, 4F, S2D, and S2E). The effect of iSPN stimulation was less pronounced than of dSPN stimulation (Figure 4D).

Are the changes in cortical activity a direct effect of optogenetic stimulation or an indirect effect of altered lick responses? To address this question, we applied linear models^55^ (see STAR Methods and Figure S3A). For the different factors (optogenetic stimulation, licks, visual stimulus) we estimated ΔR^2^, a measure of uniquely explained variance (Figures S3B–S3E). Although the visual stimulus explained significantly more variance than optogenetic stimulation (repeated measures ANOVA with variables factor and area, significant interaction: F_12,132_ = 5.39, *p* < 0.001, Bonferroni corrected t-tests indicated in Figures S3C–S3E), optogenetic stimulation still explained a significant amount of variance that only decreased after stimulus onset (Bonferroni corrected t-tests across mice, ΔR^2^>0, *p* < 0.05). Licks explained a significant amount of variance in area M2 (Bonferroni corrected t-tests across mice, ΔR^2^>0, *p* < 0.05), but only after stimulus onset (Figures S3C–S3E).

These results, taken together, demonstrate that direct pathway stimulation causes a relative widespread increase of cortical activity and that indirect pathway stimulation only briefly and locally changes cortical activity in the visual detection task. The observed activity changes are explained by both the influence on the lick responses and the direct effects of dSPN stimulation on cortical activity.

### Persistent neuronal activity during a delayed response task

**Figure 5:**
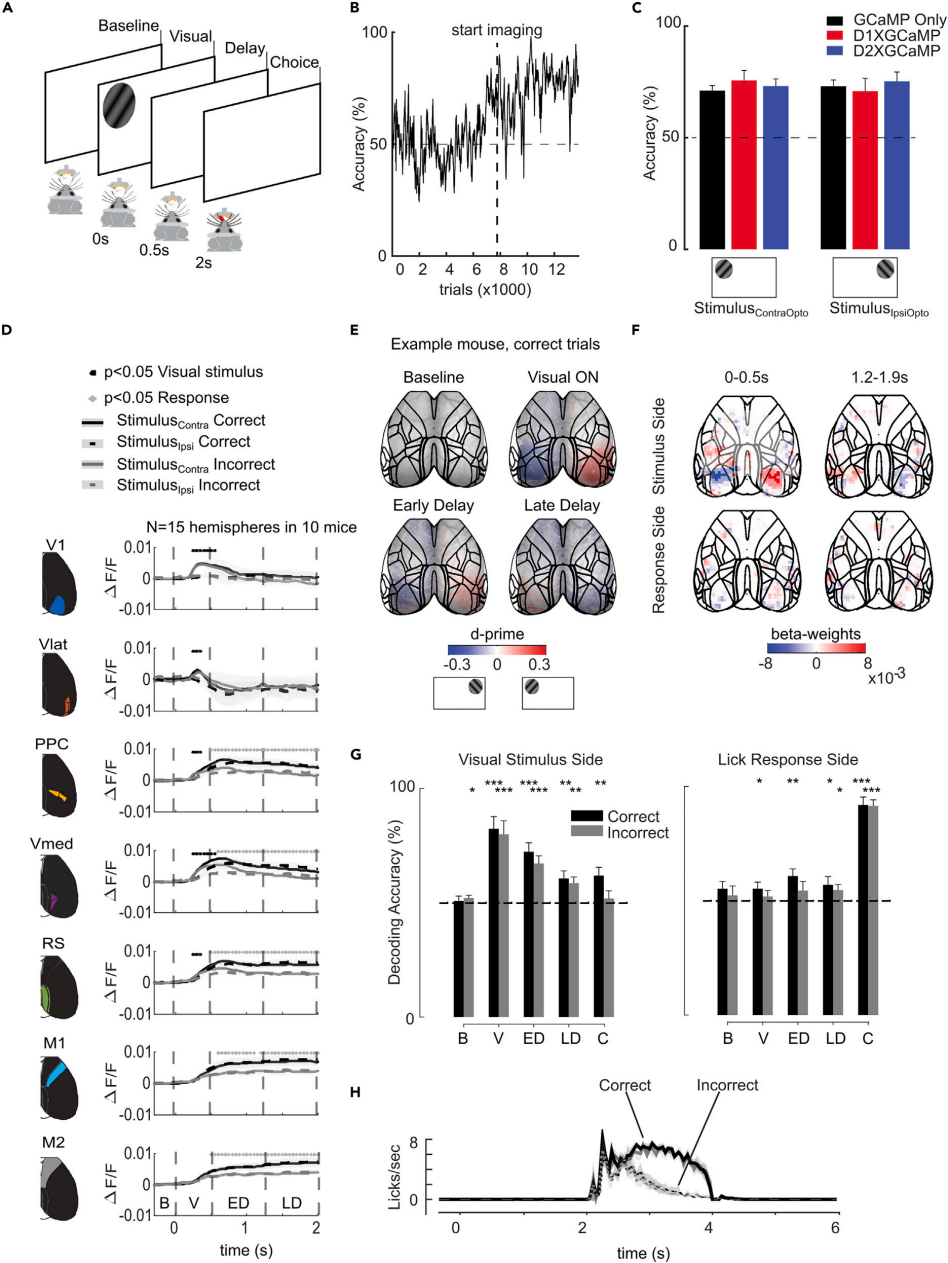
Cortical activity in the delayed response task (A) Delayed response task. We presented a drifting grating in the left or right hemifield for 500ms. After a delay of 1500ms, a two-sided spout was placed close to the mouth of the animal to encourage a lick response. Licks on the same side as the stimulus were rewarded. (B) Learning curve of an example mouse. (C) Average ± s.e.m. accuracy (black: GCaMP only, red: D1-cre x GCaMP, blue: D2-cre x GCaMP). (D) Time-course of ΔF/F ± s.e.m. in ROIs. Black (gray) traces, correct (erroneous) trials. Solid (dashed) traces show activity elicited by a stimulus in the contralateral (ipsilateral) hemifield. Dashed vertical lines indicate the start of different epochs: baseline, visual stimulus presentation, early and late delay. Black lines above the graph show significant main effects of stimulus side, gray lines a main effect of correct vs. error trials in a two-way ANOVA per time point. Note the higher activity in medial and anterior regions for correct trials. (E) D-prime for the side of the stimulus in successive time windows for an example mouse. Colored regions denote significant d-prime values (t-test, *p* < 0.05, uncorrected). The fluorescent image of this mouse is shown in gray in the background. (F) Beta-weights of the MALSAR decoder for side of stimulus (upper panels) and upcoming lick direction (lower panels) in the visual and late delay time window (0-0.5s and 1.2–1.9s after visual stimulus onset). Red pixels are positively correlated with the stimulus in the left hemifield (stimulus_ContraOpto_) or left lick (lick_ContraOpto_) and blue pixels with the other stimulus and lick direction. Opaque pixels show beta-values significantly different from zero across mice (*N* = 10, t-test, *p* < 0.05, uncorrected). (G) Cross-validated decoding accuracy (mean ± s.e.m.) across mice (*N* = 10) for side of the visual stimulus (left panel) and lick response (right panel). Time-windows: baseline (B), visual (V), early delay (ED), late delay (LD), and choice (C). Black (gray) bars represent correct (erroneous) trials. Decoding accuracy for stimulus and lick response side depended on the time-window (ANOVA with factors correctness and time window, for stimulus position decoding: F_4,90_ = 22.1, *p* < 0.001 and for lick direction: F_4,90_ = 56.3, *p* < 0.001), but not on the trial outcome (correct/error). ∗, *p* < 0.05; ∗∗, *p* < 0.01; ∗∗∗, *p* < 0.001 in post-hoc t-tests, testing whether decoding was better than chance level (0.5). (H) Average lick frequency (licks/sec; shade regions shows s.e.m. across mice) for correct (solid) and incorrect (dashed) responses, for visual stimuli presented in the left (black) and right (gray) hemifield. The lick pattern diverged for correct versus incorrect trials after 2.2s, presumably because of the water reward. The lick responses started after the epoch (>2s) in which we analyzed neural data (0-2s).

Our last experiment explored the influence of dSPNs and iSPNs in a task that included a memory interval and a delayed motor response. Specifically, the mice carried out a two-alternative-forced-choice task with a 1.5 s delay between the visual stimulus and the motor response (Figure 5A). The visual stimulus (same as described above) was presented on the left or right side of the screen and the mice had to report the location of the visual stimulus by licking the corresponding side of the two-sided lick spout. In this version of the task, a reward was only given if the first lick was on the same side as the visual stimulus. Most mice (77%) performed the task above chance level after 40–100 training sessions (5 training sessions per week), with 150–250 trials per session (Figures 5B and 5C; left stimulus: 73.6 ± 2.4% and right stimulus: 73.0 ± 6.3; *p* < 0.001 for both visual stimuli). Mice that did not learn the task were excluded from further analysis (10 mice remained). In this task, the lick responses occurred after 2s, and we analyzed the neuronal activity in the preceding time interval (Figure 5H).

In accordance with previous work, the task activated regions of the visual and motor cortex.^9^^,^^19^^,^^56^ We analyzed the results with a repeated measures ANOVA for every time point, with factors correct/error and visual stimulus position across hemispheres (*N* = 15 hemispheres). As expected, the visual stimulus initially drove activity in the contralateral visual cortex (ps < 0.05 for at least 150ms; Figures 5D and 5E). In the delay epoch, we observed persistent activity in many cortical areas. Persistent activity was higher on correct trials, particularly in frontal ROIs (ps < 0.05 for at least 150ms; compare black to gray traces in Figure 5D), suggesting that engagement in the task boosts persistent activity.

Next, we examined how delay activity encoded the position of the visual stimulus. In the early phase of the delay epoch (0-750ms after the stimulus had disappeared), activity remained stronger contralateral to the visual stimulus in V1 (Figures 5D and 5E). Interestingly, in a later phase of the delay (750-1500ms after stimulus offset) the pattern inverted but this trend was not significant across hemispheres (ps > 0.05; repeated measures ANOVA for every time point). Overall, the delay activity in individual areas was not consistently related to the memory for the position of the visual stimulus. We considered the possibility that the absence of this relation might have been caused by variability across mice.

To account for potential differences between mice, we used a multi-output decoding approach (MALSAR) to decode stimulus position and the upcoming licking direction from cortex-wide activity^57^ (Figure S4A). We balanced the trials so that there was an equal number of the four combinations of correct/incorrect responses and left/right visual stimuli. In line with the inversion described above (Figures 5D and 5E), the decoding weights in visual areas inverted in the late delay epoch (Figure 5F). The accuracy of decoding varied across trial time (Figure 5G) (*N* = 10 mice, ANOVA with factors correctness and time window, for stimulus position decoding: F_4,90_ = 22.1, *p* < 0.001 and for lick direction: F_4,90_ = 56.3, *p* < 0.001). Decoding of the stimulus was best while it was visible and the accuracy decreased with time, although it was still better than chance in the delay epoch (Figure 5G, left panel, *p* < 0.01 post-hoc t-tests). Accuracy of decoding the upcoming lick direction increased during the trial and was close to 100% during the response (Figure 5G, right panel, *p* < 0.05 post-hoc t-tests). Taken together, these results indicate that persistent activity reflects the previously presented stimulus as well as the upcoming lick response, suggesting that it signals a working memory.

### The direct and indirect pathways influence accuracy during the delay-task

We tested the influence of optogenetic stimulation on accuracy in two D1-cre-mice and three D2-cre-mice in the delay-task. Without optogenetic stimulation, the accuracy of these mice was comparable to that of the control mice (Figure 5C). For each trial in which we applied optogenetic stimulation, it was in one of the following epochs: just before the visual stimulus (−0.5-0s), during the visual stimulus (0-0.5s), the delay (1-1.5s), the response epoch (2-2.5s) or in the ITI (3.5-4s).

**Figure 6:**
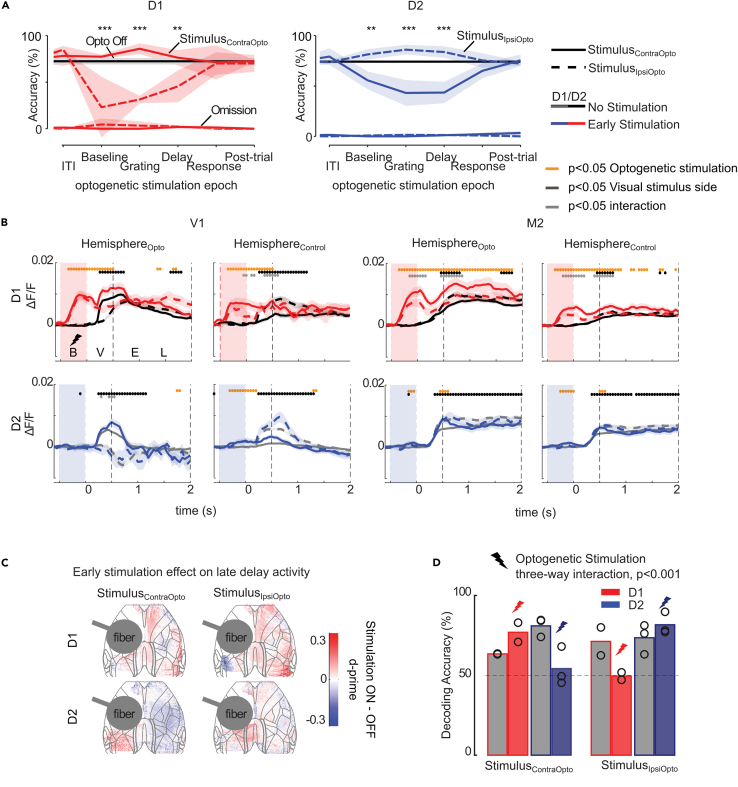
Optogenetic stimulation of the striatum in the delayed response task (A) Influence of optogenetic stimulation in different time-windows (x axis) on the accuracy ± s.e.m. of D1-cre (left panel) and D2-cre (right panel) mice. The black horizontal line shows accuracy in trials without optogenetic stimulation. Blue and red solid (dashed) lines show accuracy (top lines) and percentage omission (bottom lines) for stimulus_ContraOpto_ (stimulus_IpsiOpto_). Optogenetic stimulation did not alter the percentage of omissions. Accuracy increased (decreased) for stimulus_ContraOpto_ (stimulus_IpsiOpto_) in D1-cre mice, and the opposite was true for D2-cre mice. ∗∗∗, *p* < 0.001; ∗∗, *p* < 0.01 reflect differences between stimulus_IpsiOpto_ and stimulus_ContraOpto_ (post-hoc tests after mixed linear model). (B) Influence of optogenetic stimulation (colored traces) in the baseline period (from -500-0ms) on M2 and V1 activity. Traces show average ΔF/F and shaded regions s.e.m. Solid (dashed) lines represent trials with stimulus_ContraOpto_ (stimulus_IpsiOpto_). Mixed-effects models per time point showed significant main effects of optogenetic stimulation (orange circles, *p* < 0.05), visual stimulus side (black circles, *p* < 0.05) and an interaction between these factors (gray circles, *p* < 0.05) (see Figure S5 for optogenetic stimulation during the visual stimulus presentation). (C) D-prime of the optogenetic effect (pooled across all early optogenetic stimulation trials) during the late delay (1200-1900ms after stimulus onset). (D) We trained a model to decode stimulus position based on cortical activity during the late delay on trials without optogenetic stimulation. We then tested the model on late delay cortical activity of trials with early optogenetic stimulation (red/blue). We also included a separate test-set of trials without stimulation (gray). Depicted is the percentage of trials that the model predicted the memory representation of stimulus_ContraOpto_ (left) and stimulus_IpsiOpto_ (right) correctly. The increase in accuracy for stimulus_ContraOpto_ for D1-cre mice (bars with red outline) after early optogenetic stimulation of the striatum shows that cortical activity in the late delay is biased to represent stimulus_ContraOpto_, and vice versa for D2-cre mice (bars with blue outline). A three-way ANOVA revealed a significant interaction between optogenetic stimulation, visual stimulus side and genotype (*p* < 0.001) (see Figure S6 for more details). Circles represent decoding accuracy for individual mice.

The influence of dSPN stimulation on accuracy depended on the position of the visual stimulus, the epoch of stimulation, and the genotype of the mouse (mixed effects model, significant interaction between epoch, stimulus side, and genotype: F_6,42_ = 18.7, *p* < 10^−9^). Specifically, stimulation of dSPNs during the visual stimulus presentation and delay epochs increased contraversive choices, boosting the accuracy for stimulus_ContraOpto_ and decreasing it for stimulus_IpsiOpto_ (post-hoc Wald tests of coefficients, ps < 0.01, Figure 6A). Interestingly, this effect also occurred when we applied optogenetic stimulation just prior to the visual stimulus. Stimulation of iSPNs in D2-cre-mice had the opposite effect on accuracy by increasing the number of ipsiversive choices. The difference in accuracy between stimulus_IpsiOpto_ and stimulus_ContraOpto_ was significant with stimulation in the pre-stimulus epoch, during the visual stimulus and during the delay. Stimulation of dSPNs and iSPNs did not influence the number of omission trials without a lick response (Figure 6A) (mixed effects model as above, but with omission as predicted variable, all ps > 0.05). In this task, the mice had been trained extensively to withhold their lick response until the response window (see STAR Methods). We did not observe licks induced by optogenetic stimulation outside the response epoch. Hence, activation of the direct and indirect pathways prior to the late delay had a pronounced influence on the decision that was taken at the end of the trial.

### The direct and indirect pathways influence neuronal representations of working memory

To analyze the influence of direct and indirect pathway stimulation on cortical activity during the delay-task, we combined all trials with a behavioral effect, prior to the late delay phase of the trial (Figure 6A). Specifically, we included trials in which the optogenetic stimulation was given in the (pre-stimulus) baseline epoch (Figure 6B), during the presentation of the visual stimulus and early in the delay.

dSPN stimulation caused a rapid increase in global cortical activity (see Figure 6B for two example ROIs, V1 and M2). Interestingly, optogenetic stimulation early in the trial had an effect on cortical activity at time points later in the trial (mixed effects model performed per time point: orange symbols in Figure 6B show a main effect of optogenetic stimulation and gray symbols the interaction with visual stimulus position, *p* < 0.05, see also Figure S5). Activation of dSPNs increased the activity in M2 in both hemisphere_Opto_ and hemisphere_Control_, although the increase in hemisphere_Opto_ was more pronounced. Activation of iSPNs stimulation initially increased the visually driven response in visual cortex, but during the late delay we observed a slight decrease in persistent activity for stimulus_ContraOpto_ (Figures 6B and 6C, S5). We next asked whether the effect of optogenetic stimulation on the decision (Figure 6A) was caused by an altered memory of the stimulus or by an effect on the upcoming lick response.

To examine the influence of the striatal pathways on the memory representation of the visual stimulus, we trained decoding models using cortical activity in the late delay on trials without optogenetic stimulation, balancing the number of trials in the different conditions as described above. We then inferred how optogenetic stimulation early in the trial influenced predictions during the late delay, by pooling trials with stimulation during the baseline epoch, the visual stimulus and the early phase of the memory delay (Figure S4B). Note that in the late delay epoch there is no direct contribution from either optogenetic stimulation or lick responses. Without optogenetic stimulation, decoding accuracy for the location of the visual stimulus was significantly higher than chance level in all mice (gray bars in Figure 6D). Early dSPN stimulation caused a bias of the decoder toward output stimulus_ContraOpto_. In contrast, early iSPN stimulation biased the decoder to output stimulus_IpsiOpto_ (ANOVA, significant interaction of the three factors optogenetic stimulation, visual stimulus side and genotype: F_1,12_ = 20.2, *p* < 0.001, Figure 6D, see Figure S6C for data of individual mice). This result indicates that optogenetic stimulation of the direct and indirect pathways influenced the memory representation of the visual stimulus, irrespective of lick-direction or measured body movements (Figure S6C).

Even though the optogenetic stimulation impacted the probability of choosing left versus right, it did not impact on the decoding of the choice (Figures S6B–S6D), indicating that the relation between cortical activity and the upcoming choice was unaltered. These results, taken together, suggest that optogenetic stimulation altered the memory representation of the stimulus and influenced the upcoming choice, without a change in the relation between licking direction and cortical activity as measured through wide-field imaging.

## Discussion

We investigated the influence of direct and indirect pathways of the basal ganglia on perceptual decisions and activity in the cerebral cortex. In the absence of a task, dSPN stimulation led to a global increase of cortical activity, whereas stimulation of iSPNs had a weak and more local effect. In the visual detection task, stimulation of dSPNs increased the number of contraversive licks (Figure 4). It also caused an overall increase in cortical activity, which was largest if the visual stimulus fell in the hemifield contralateral to hemisphere_Opto_, an interaction that was most pronounced in the more anterior ROIs. In contrast, iSPN stimulation increased the number of ipsiversive licks and it decreased neuronal activity in the visual cortex of hemisphere_Opto_. In our final experiment, we tested the influence of dSPNs and iSPNs in a delayed response task. We first established signatures of cortical activity that were associated with the visual stimulus, its memory, and the direction of the lick response. We then applied optogenetic stimulation to the striatum and found that direct pathway activation increased the number of future contraversive choices. This effect was observed with optogenetic stimulation in the epochs just before the stimulus appeared, during the presentation of the visual stimulus, and during the delay between stimulus and lick response, but not in epochs seconds before the trial or during the lick response itself. Dorsal cortical activity at the end of the delay period was modulated in line with these behavioral changes.

We used an MALSAR decoder with a balanced number of trials per condition to determine the relation between cortical activity, behavioral choice and the location of the visual stimulus. Using this analysis, we found that dSPNs stimulation during earlier time points caused cortical activity to resemble a working memory of a visual stimulus presented contralateral to hemisphere_Opto_, in accordance with the increase in contraversive choices. In contrast, iSPN activation in the working memory task increased future ipsiversive choices, an effect that was associated with a cortical activity pattern resembling the working memory of a visual stimulus ipsilateral to hemisphere_Opto_. In contrast, we did not observe a strong influence of optogenetic stimulation on choice decoding. Hence, optogenetic stimulation altered the memory representation of the stimulus and influenced the choice, but it did not influence the relation between cortical activity and lick direction.

Previous studies have linked the basal ganglia to the execution of motor sequences and the mapping of stimuli onto responses^58^^,^^59^ through reinforcement learning.^60^ Indeed, the influence of the dorsal striatum on behavior depends on the task and the actions that are associated with a reward.^21^^,^^43^^,^^61^ We found that the direct and indirect pathway in the striatal region that we targeted (the ventromedial part of the dorsal striatum) promote contraversive and ipsiversive licks, respectively. The effect of dSPN and iSPN activation depended on the location of the visual stimulus^62^ and on the timing relative to the visual stimulus. We attribute these influences to the mapping of visual stimuli onto motor responses. In other words, this striatal region does not solely instruct lick responses at the motor level and, accordingly, the mice could switch to the other direction if their first lick was in the wrong direction, in spite of optogenetic activation.

Our findings in the detection task support findings by Lee et al. (2020),^24^ who trained mice to report one of two auditory tones with a left or right lick response. In their experiment, dSPN stimulation also increased contraversive licks, which was associated with more activity in the region of the motor cortex related to licking responses. They recently demonstrated that iSPNs influence activity in the superior colliculus,^43^ which also influences licking.^63^ The iSPNs inhibit the ipsilateral and excite the contralateral superior colliculus, thereby increasing ipsiversive licks. Similarly, dSPNs excite the motor cortex, whereas iSPNs have an inhibitory influence.^64^ Our results are also in line with the findings of Tai et al. (2012)^65^ who showed that transient stimulation of the striatal pathways biases decisions in the near future. Yet, our results go beyond on these previous studies by demonstrating an effect on the working memory of a visual stimulus (Figure 6D) and on lick responses more than a second later (Figure 6A). Our findings also complement a previous study using fMRI in anesthetized mice, which demonstrated largely opposite effects of direct and indirect pathway stimulation on many brain structures.^66^ Here, we used widefield imaging to monitor cortical activity in awake mice across several tasks, allowing us to relate the effect of the striatum on activity in the cerebral cortex to its influence on behavior.

A recent study demonstrated that the behavioral effects of inhibition of the direct and indirect pathways of the dorsomedial striatum are most pronounced in tasks with a memory requirement.^62^ In accordance with these results, we observed strong effects of striatum stimulation in the delay-task. Intriguingly, dSPN stimulation made the cortical activity pattern during the delay similar to a memory of a stimulus presented contralateral to the stimulated hemisphere,^67^ whereas iSPN activation changed cortical activity in the opposite direction.

Our results contribute to a growing body of evidence that the ventromedial part of the thalamus and the substantia nigra pars reticulata, which are part of the cortico-striatal loop, are causally involved in working memory. In a task in which mice judged the amplitude of a whisker stimulus, unilateral activation and deactivation of these nuclei during a memory delay had opposite effects on lick-direction.^22^ Here we observed opposite effects of dSPNs and iSPNs in a task in which mice memorized a visual stimulus. Indeed, sustained activity can be found in the delay period of working memory tasks in all areas involved in this loop (this study and e.g.,^9^^,^^19^^,^^21^^,^^23^^,^^24^). One exciting possibility is that the direct pathway maintains the memory, because the loop with its two inhibitory connections can cause neurons in a cortical area to provide positive feedback to themselves. Under this model, the indirect pathway, with its three inhibitory connections, may play a role in forgetting items in working memory that are no longer needed by decreasing activity in the feedback loop of the direct pathway. A study by Tecuapetla et al. (2016)^41^ provided some evidence for this view. They trained mice to make a series of lever presses for a food reward. Stimulation of dSPNs caused, under some conditions, an increase in the number of presses, prolonging the motor program. Stimulation of iSPNs, on the other hand, caused the mice to abort the task and start doing something else, as if they completely forgot the motor program in which they were engaged.

Interestingly, the striatum appears to not only influence working memory, but also the distribution of attention. Wang et al. (2018)^21^ trained mice to direct attention to one of two stimuli in a change detection task. dSPN activation caused a shift of attention to the stimulus in the contralateral hemifield. Attended stimuli are more likely to be remembered than non-attended ones,^68^^,^^69^ and it is therefore probable that overlapping circuits of the basal ganglia contribute to attention shifts and working memory.

The present results support the view that immediate and delayed perceptual decisions are, in part, mediated by the loop between the cortex and the basal ganglia – although loops between cortex, cerebellum, and the thalamus may also play a role.^70^ Persistent neuronal activity related to working memory appears to be an emergent property of distributed networks^11^^,^^18^ where the striatal pathways orchestrate cortical activity by integrating sensory and motivational inputs to support adaptive behavior.

### Limitations of the study

Our working memory task was not designed to tease apart the various processes that map visual stimuli onto motor responses. To gain more insight into these stages, it would be of interest to test a reversal version of the task in which mice have to lick in the opposite direction as the visual stimulus. However, we did not pursue this reversal version given the preference of mice to lick on the same side as the visual stimulus^71^ and that it already took minimally eight weeks to train mice on the congruent version of the task. We used an MALSAR decoder with a balanced number of trials per condition to determine the relation between cortical activity, behavioral choice, and the location of the visual stimulus. Taken together, our results show that optogenetic stimulation altered the memory representation of the stimulus and influenced the choice, but it did not influence the relation between cortical activity and lick direction.

Lee et al. (2016)^66^ found robust responses to optogenetic stimulation of both D1 and D2 neurons. In contrast, we only observed strong neuronal responses to direct pathway stimulation and only weak effects of indirect pathway stimulation during ‘resting state’ and local cortical effects during the tasks. There are several potential reasons for this discrepancy. The first is a difference in expression between D1-cre and D2-cre mice, although we note that the expression patterns were similar in our experiments (Figure 1). The second is a difference between optogenetic stimulation protocols. We used a pulse train with a duration of 1s, whereas Lee et al. (2016) used a pulse train of 20s. It is conceivable that prolonged stimulation of the indirect pathway enhances the modulation of cortical activity. A third difference is that we tested awake mice whereas Lee et al. (2016) used anesthesia which has a profound influence on functional connectivity.^72^ Finally, the number of mice in some of the tasks was limited, but we nevertheless obtained robust effects of optogenetic stimulation.

## Resource availability

### Lead contact

Further information and requests for resources and reagents should be directed to and will be fulfilled by the lead contact, E.v.B. (ennyvanbeest@gmail.com).

### Materials availability

This study did not generate new unique reagents.

### Data and code availability

• Example data to regenerate figures will be shared via an online repository as of the date of publication. Zenodo: https://doi.org/10.5281/zenodo.13235302. Other preprocessed data reported in this paper will be shared by the lead contact upon request.
• Original code has been deposited at https://github.com/EnnyvanBeest/vanBeest_iScience2024 and is publicly available as of the date of publication. URLs are listed in the key resources table.
• Any additional information required to reanalyze the data reported in this paper is available from the lead contact upon request.

## Acknowledgments

We thank the animal and mechatronics departments at the Netherlands Institute for Neuroscience for their assistance. We thank Areg Barsegyan, Ulf Schnabel, Thijs Baaijen and Cédric Gillissen for their assistance in setting up widefield imaging and analysis. We thank Roxana Kooijmans for advice on histology, Emma Ruimschotel and Christiaan Levelt for support with breeding, Chris van der Togt, Mustafa Hamada, Ralph Hamelink and Nicole Yee for technical assistance, Kor Brandsma, Bastijn van den Boom and Sreedeep Mukherjee for surgical assistance and advice and Rudolf Faust, Chris Klink and Lisa Kirchberger for help and advice throughout the experiments. This work was supported by the European Union’s Horizon 2020 Framework Programme for Research and Innovation under the Framework Partnership Agreement No. 650003 (HBP FPA), 10.13039/100010664FET Open grant number 899287 ‘NeuraViPeR', Crossover grant number 17619 ‘INTENSE’ from 10.13039/501100003246NWO and an NWO-Klein grant OCENW.KLEIN.178.

## Author contributions

E.v.B. developed the behavioral tasks and set-up combined widefield imaging and optogenetics. E.v.B., with help from CM, performed the visual detection experiments combined with optogenetics. E.v.B., with help from C.B., performed the delayed response experiments in control mice. E.v.B., with help from M.A.O.M., L.C., and B.P. performed the combined widefield and optogenetic experiments. E.v.B. analyzed the data with input from M.W.S. and P.R.R. E.v.B., with help from C.M., C.B., L.C., and B.P. performed histology. E.v.B., P.R.R., and I.W. conceived of the experiments, and P.R.R., I.W., and M.W.S. supervised the project. E.v.B. wrote the paper with help from M.W.S., I.W., and P.R.R. All remaining authors reviewed the manuscript except for MAOM.

## Declaration of interests

The authors declare no competing interests. Affiliations to other research institutions that authors may have at the time of publication are not relevant for the work in this manuscript, nor do they form a conflict of interest.

## STAR★Methods

### Key resources table

**Table:** 

REAGENT or RESOURCE	SOURCE	IDENTIFIER
**Bacterial and virus strains**		

AAV5-Syn-Flex-rcChrimsonR-tdTomato	Edward Boyden (Klapoetke et al. 2014^73^)	RRID:Addgene_62723
AAV1 EF1a DIO hChR2(H134R) eYFP WPRE hGH	Karl Deisseroth	RRID:Addgene_20298(AAV1)

**Deposited data**		

Preprocessed data	This paper	Zenodo: https://doi.org/10.5281/zenodo.13235302

**Experimental models: Organisms/strains**		

Thy1-5.17 GCaMP6f (C57BL/6J-Tg(Thy1-GCaMP6f)GP5.17Dkim/J)	Dana et al. 2014^52^	RRID:IMSR_JAX:025393
Drd1-cre (B6.FVB(Cg)-Tg(Drd1-cre)EY262Gsat/Mmucd)	Nathaniel Heintz, Ph.D., The Rockefeller University, GENSAT and Charles Gerfen, Ph.D., National Institutes of Health, National Institute of Mental Health	RRID:MMRRC_030989-UCD
Drd2-cre (B6.FVB(Cg)-Tg(Drd2-cre)ER44Gsat/Mmucd)	Nathaniel Heintz, Ph.D., The Rockefeller University, GENSAT and Charles Gerfen, Ph.D., National Institutes of Health, National Institute of Mental Health	RRID:MMRRC_032108-UCD
Thy1-5.17 GCaMP6f x Drd1-cre	This paper	N/A
Thy1-5.17 GCaMP6f x Drd2-cre	This paper	N/A

**Software and algorithms**		

MATLAB	Mathworks	https://mathworks.com/
Cogent toolbox	John Romaya at the LON	https://www.ucl.ac.uk/∼ucgajpr/contents.html
Encephalos software package	Caenotec – Ralf Schnabel	http://www.simi.com/download/biocell/4D_Handbook_21_09_2010.pdf
AP_Histology	Andrew Peters	http://github.com/petersaj/AP_histology
MALSAR: Multi-task learning via structural regularization	Zhou et al., 2012^57^	https://jiayuzhou.github.io/MALSAR/
Modeling the spatiotemporal dynamics of light and heat propagation for *in vivo* optogenetics	Stujenske et al., 2015^74^	https://www.cell.com/cell-reports/fulltext/S2211-1247(15)00648-8#secsectitle0135
MATLAB Toolbox emmeans	Hartman, 2019^75^	https://github.com/jackatta/estimated-marginal-means
Behavioral and imaging analysis code	This paper	https://github.com/EnnyvanBeest/vanBeest_iScience2024

### Experimental model and study participant details

We included 6 Thy1-5.17 GCaMP6f mice,^52^ 8 D1-cre, 10 D2-cre, 9 Thy1-5.17 GcaMP6f X D1-cre, and 7 Thy1-5.17 GcaMP6f X Drd2-cre of mixed sex, aged 2–6 months at the start of the experiment (Table S2). Animals were housed either in pairs or in isolation and kept on a 12 h/12 h reversed day/night cycle. Experiments were performed in the dark phase. All experimental procedures complied with the National Institutes of Health Guide for Care and Use of Laboratory Animals and the study protocol (NIN 18.18.03) were approved by the ethical committee of the Royal Netherlands Academy of Arts and Sciences and the CCD. The experiments were performed in accordance with all relevant guidelines and regulations.

### Method details

#### Surgery – Preparation

Anesthesia was induced using 3–5% isoflurane in oxygen enriched air (50% air, 50% O_2_) in an induction box. The mice were positioned in a stereotactic frame and the depth of anesthesia was monitored throughout the surgery by frequently checking paw reflexes and breathing rate and the concentration of isoflurane was adapted accordingly (between 0.8 and 2.5%). We subcutaneously injected 5 mg/kg meloxicam (0.5 mg/mL) as general analgesic. We monitored the temperature of the animal and kept it between 36.5° and 37.5° with a heating pad coupled to a rectal thermometer. Eyes were covered with ointment to prevent dehydration. The area of incision was shaved, cleaned with alcohol and betadine or hibicet, and lidocaine spray was applied to the skin as local analgesic. An incision was made in the skin along the anteroposterior midline, exposing the skull above the cortex and posterior to lambda. The periost and other tissue was removed from the skull by scraping, rinsing and briefly applying H_2_O_2_ or hibicet.

#### Surgery – Clear skull and head plate

Once the dorsal skull was completely exposed and dry, a thin layer of adhesive (cyanoacrylate glue Bison) was applied to the bone, thereby making the bone transparent. This effect occurs over the course of the following days and is referred to as the “clear skull cap” technique.^54^ A thin layer of clear dental cement (C&B super-bond) and nail polish (Electron Microscopy Sciences) were applied for strengthening and to reduce light glare during imaging. A platform of dental cement (Heraeus Charisma) was built posterior to lambda to place the head-bar (for head-fixation purposes). Multiple layers of cement were used to secure the head-bar on the skull. On the outer edges of the clear skull, a small wall of cement (Heraeus Charisma) was built to prevent the skin from growing over the area of interest. The mice were monitored and kept warm while recovering from anesthesia. The mice had a minimum of two days to recover before they were habituated to set-ups and trained.

#### Surgery – Optogenetics

In D1/2-cre positive mice we additionally performed two craniotomies. The first was for the injection of the virus in the right hemisphere, at 2.5 ML, +0.14 AP from bregma. A glass pipette with AAV5-Syn-Flex-rcChrimsonR-tdTomato^73^ (a gift from Edward Boyden - Addgene #62723, 160nL in total, 1.1∗10ˆ13 GC/mL, diluted 1:0.5–2 in saline) or AAV1 EF1a DIO hChR2(H134R) eYFP WPRE hGH (a gift from Karl Deisseroth - Addgene #20298-AAV1, 160nL in total, 2.216∗10ˆ13 GC/mL, diluted 1:0.5–2 in saline) was lowered to a depth of -4mm relative to bregma. A total of 160nL was injected in 8 pulses of 10 nl/s, with 30 s in between pulses. The second craniotomy was made over the left hemisphere, 2mm lateral and 0.14 anterior to bregma. A custom-made glass optical fiber (200micron diameter, 0.39NA, ∼5.5mm long) was inserted in an angle of 48°, such that the tip of the glass-fiber would end ∼1mm above the center of virus injection. The ferrule was secured with dental cement (Heraeus Charisma and C&B super-bond). After securing the ferrule the clear-skull procedure was applied.

#### Visual stimuli

Visual stimuli were created using the Cogent toolbox (developed by John Romaya at the LON at the Wellcome department of Imaging Neuroscience) and the luminance profile of the monitor was linearized. Some mice included in Figure 4B were positioned 11cm (from the eye) in front of a 24-inch LCD monitor (1920 x 1200 pixels, Dell U2412M) (Table S2). All other experiments were done in the wide-field imaging set-up with a different LCD monitor (122 × 68cm, liyama LE5564S-B1). These mice were positioned at a distance of 14cm from the screen. We applied a previously described correction for the larger distance between the screen and the mouse at higher eccentricities.^76^ This method defines stimuli on a sphere and calculates the projection onto a flat surface. Figures were comprised of 100% contrast sinusoidal gratings, with a diameter of 35°, 0.08 cycles/° and mean luminance of 20 cd/m^2^. The gratings appeared on the screen at an eccentricity of 40°and 15° elevated relative to the nose of the mouse. Oriented figures (45° for figures on the left side and 135° for figures on the right side) were presented on a gray background (20 cd/m^2^), moving with a speed of 24deg/s in a direction orthogonal to the orientation.

#### Training mice on the behavioral tasks

We handled the mice daily for 5–10 min before we started the training protocol.^77^ Head-fixation training started by holding the head-bar for a few seconds in the home cage. After one or two days, the animals were head-fixed daily for an increasing amount of time, until they were accustomed to being head-fixed on the set-up. At this point, the animals were put on a fluid restriction protocol with a minimal intake of 0.025 mL/g per day, while their health was carefully monitored. First, animals (except Thy1-GcaMP animals, which were only trained on the two-alternative forced choice task, described below) were trained to indicate the appearance of a visual stimulus (visual detection task) by licking either side of a custom-made double lick-spout (Figure 4A). Licks were registered by measuring a change in capacitance with an Arduino using custom-written software. A lick to either side of the lick spout was counted as correct and rewarded with 5-8μL of water or milk (Nutrilon). Stimuli on the left were followed by fluid delivery on the left and stimuli on the right by fluid delivery on the right. To prevent mice from licking continuously, they had to withhold licking for a period of 2–4 s (uniform distribution) prior to the start of a trial.

Some animals received additional training in a two-alternative-forced-choice (2AFC) task after they completed the visual detection task. In this task they had to indicate the side on which a figure appeared by licking the corresponding side of the double lick-spout. A trial started after a variable inter trial interval (ITI) of 6–10 s when the stimulus appeared on the screen for 500ms. Now correct responses required the first lick on the side of the visual stimulus. If the animal made an error, a 5s timeout was added to the ITI. We gradually increased the task-difficulty by increasing the delay between stimulus and response from 0 to 1500ms over several weeks of training, based on performance (staircase). In the final delayed response task, mice had to withhold a lick-response for 2000ms (including the 500ms stimulus time). The delay was enforced by attaching the lick-spout to a servo motor (Arduino). The servo motor moved the lick-spout to a position that mice were still able to reach, but at a distance that was not comfortable. Most mice quickly learned that licking the spout at this distance did not yield reward. Other mice persevered premature licking behavior even with the lick-spout at some distance. For these animals we aborted trials immediately after premature lick-responses or body movement (measured with a piezo element) to discourage this behavior. We monitored the right pupil (50-100Hz sampling rate) and extracted the size and position of the pupil online using custom built tracking software. Movements of the mouse were measured with a piezoelectric sensor fixed to the head-fixation plateau, situated under the front paws (100Hz sampling rate), amplified and relayed using custom-built Arduino (software/hardware). This data was stored as ‘pressure’ with the extracted eye-data. These behavioral data were aligned and down sampled to the frequency of imaging acquisition using triggers that were stored in both the widefield images as well as the behavioral files. Behavioral data were further processed as eye and body movements: the absolute difference between two subsequent timepoints. Normalized eye (averaged over changes in width, height, x- and y-position of the pupil) and body movements were included in the linear models (see STAR Methods) and we examined the movements during the behavioral task (Figure S6E). A time-out of 5 s followed incorrect lick-responses.

#### Wide field imaging

After habituation to the set-up, mice were placed under a wide-field fluorescence microscope (Axio Zoom.V16 Zeiss/Caenotec) to image a large part of the cortical surface. Images were captured at 20Hz by a high-speed sCMOS camera (pco.edge 5.5) and recorded using the Encephalos software package (Caenotec). We monitored size and position of the right pupil (50-100Hz sampling rate) and movements of the mouse with a piezo plate under the front paws (100Hz sampling rate). Eye and body movements were included in the linear models.

#### Optogenetics

For optogenetic stimulation of the striatum we used a fiber-coupled DPSS Laser (Shanghai Lasers & Optics Century Co.) emitting blue light (BL473T3-100FC, wavelength 473nm) for mice injected with ChR2, and red light (RLM638TA, wavelength 638nm) for mice injected with ChrimsonR. Based on data from our pilot experiments and previous studies we used 15Hz stimulation with a 10ms pulse width.^40^ The duration of the light train differed between experiments. During spontaneous behavior we stimulated for 1 s. In the visual detection task optogenetic stimulation occurred in 40% of trials. In 20% of trials, stimulation occurred 0.5s prior to visual stimulus onset, and in the other 20% of trials it started after the first lick following visual stimulus onset. In the delayed response task we stimulated for 500ms. The time between two successive optogenetic stimulations was at least 5 s, but usually between 8 s to a few minutes. Optogenetic stimulation during the delayed response task only occurred when the accuracy of the mice was above 65% in the preceding 15 trials, to prevent the development of lasting behavioral biases as a result of frequent unilateral optogenetic stimulation.

Light intensity was calibrated per mouse. Prior to implantation the throughput of the optic fibers was measured with a standard photodiode power sensor (S120C Thorlabs). Initially, the light intensity was 2.5 mW/mm^2^ and it was gradually increased to maximally 6.0 mW/mm^2^ if we did not observe an effect on lick-behavior in the simple detection task. These light levels do not cause measurable heating, especially if the light is delivered in pulses of 10ms.^74^^,^^78^ We included only data after the calibration was finished and when the light intensity was stable. To prevent that the light itself was perceived as a cue, the fiber was within the light-shield of the mouse that was also used for widefield imaging. In addition, a LED positioned close to the fiber but outside the light shield flashed light with a similar wavelength at random times, but with identical duration, pulse width and frequency.

#### Histology

To examine virus expression, we euthanized mice with nembutal and transcardially perfused them with phosphate buffered saline (PBS) followed by 4% paraformaldehyde (PFA) in PBS. We extracted the brain and post-fixated it overnight in 4% PFA before moving it to a PBS solution. We cut the brains into 50μm thick coronal slices and mounted them on glass slides. We imaged the slices on a Zeiss Axioscan Z1 or Zeiss Axioplan 2 microscope (10x objective, Zeiss plan-apochromat, 0.16NA) using custom written Image-Pro Plus software and aligned the images in 3D to the Allen Brain common coordinate framework using a slightly adapted version of a publicly available toolbox (http://github.com/petersaj/AP_histology). We visualized the spread of the virus and determined the optic fiber tract in 3D for individual mice. We modeled the spread of the light from the fiber using software described in Stujenske et al. (2015)^74^ and only included mice with virus expression in the striatum and an appropriate placement of the fiber in the D1-cre and D2-cre groups. We excluded mice with virus expression that was too widespread. We included mice with no (visible) expression or expression in areas that were not reached by the light as controls. Average intensity maps of expression across D1-cre and D2-cre mice were generated by thresholding normalized expression levels (values larger than >99% of all values across all channels) for individual mice, and projecting this binary expression around bregma +0.14AP±550micron on a 3D aligned coronal slice at bregma +0.14AP. The intensity level of a given pixel of the coronal slice at bregma +0.14 (Figure 1E) represents the number of mice for which that pixel had expression levels above this threshold. Fiber paths were drawn in 3D for individual mice and projected in a similar way.

### Quantification and statistical analysis

#### Pre-processing of wide-field imaging data

Images were recorded at 20Hz (50ms exposure) and stored in 12-bit, 1600x1600 pixel images (∼15μm per pixel). Images were binned into 800x800 pixels and converted to 16-bit. For each session, images were semi-automatically registered (using multi-modal intensity-based translation, rotation and scaling) to a population receptive field mapping imaging session.^79^ After registration, images were smoothed using a Gaussian filter with a standard deviation of 2 pixels, and stored in a data-matrix of 400 x 400 x time x number of trials. A top-view of the Allen Brain common coordinate framework was fit to the pRF-map.^80^ We computed the average ΔF/F relative to the baseline fluorescence in a 300ms window before the visual (sessions without optogenetic stimulation) or optogenetic stimulus. We only included trials in which the accuracy of the mouse was higher than 55% for both left and right stimuli, in a window of 50 trials.

When averaging data across mice for top brain views (e.g., beta-weights in Figure 5F), each mouse’s fit to the Allen Brain common coordinate framework was aligned to one ‘template’ mouse, using an affine 2D-transformation. For results shown across brain areas (e.g., time-courses, statistics) we first averaged across pixels in each region for individual mice, based on the fit with the Allen Brain atlas before averaging across mice.

#### Statistical analysis

To analyze the imaging data, we calculated neuronal d-prime, which is a measure of how well activity (i.e., ΔF/F) differentiates between two different conditions (e.g., optogenetic stimulation versus baseline activity) on single trials.

(Equation 1) $$d\_prime=\frac{\mu_{A}-\mu_{B}\;}{\sqrt{\frac{1}{2}\left(\sigma_{A}^{2}+\sigma_{B}^{2}\right)}}$$

Here $\mu_{A}$ is the average activity for condition A, $\mu_{B}$ the average activity for condition B, and $\sigma_{A}^{2}$ and $\sigma_{B}^{2}$ the variance over trials from condition A and B, respectively.

For the memory task we calculated the accuracy, which was defined as:

(Equation 2) $$Accuracy=\frac{N_{hits}}{N_{hits}+N_{errors}}x100\%$$

and the omission percentage, which was defined as:

(Equation 3) $$Omission=\frac{N_{misses}}{N_{hits}+N_{errors}+N_{misses}}x100\%$$

We used build-in MATLAB functions to perform ANOVAs. We used mixed-effect models in the form of

(Equation 4) $$Licks\;∼\;\beta_{0}+\sum_{i=1}^{n}F_{i}*\beta_{i}+\varepsilon$$

in which *β*_*i*_ is an estimate of how much each factor (*F*_*i*_) contributes to the number of Licks. To find the best estimates (minimizing the error ε) we used MATLAB’s function fitglme. The identity of the mouse was taken into account as a random factor (i.e., random offsets for individual mice in the mixed-effect model).

For behavioral analysis related to Figures 4 and S2, we initially included genotype (D1, D2, and control), optogenetic stimulation (off and on before stimulus onset), and lick side (left and right) as factors in the mixed-effects model, assuming a Poisson distribution. To obtain the significance level of the different factors, model outputs were evaluated with an ANOVA (in MATLAB). After confirming interaction effects between these factors, we used the publicly available MATLAB Toolbox emmeans^75^ for post-hoc Wald tests of coefficients. With this complete statistical model, we confirmed that there were no effects of optogenetic stimulation on lick rate in control mice (post-hoc Wald tests of coefficients *p* > 0.05 for control mice), but only in D1 and D2 mice (*p* < 0.001). To improve readability in the main text, we then focused on separate mixed-effects models including optogenetic stimulation and lick side for D1 and D2 mice. Post-hoc tests were corrected for multiple comparisons.

To investigate whether optogenetic stimulation enforced a specific motor response, we determined the switch index (SI). The SI is defined as:

(Equation 5) $$SI=\frac{\sum C\to I-\sum I\to C}{\sum C\to I+\sum I\to C}$$

in which ∑C→I is the number of times the mouse switched from contraversive to ipsiversive licks within a trial in a time window of 1 s after the onset of the visual stimulus, and ∑I→C the number of times the mouse switched from ipsiversive to contraversive licks within a trial.

In the main results we focused our analysis on the condition where optogenetic stimulation started 0.5s prior to the visual stimulus onset. On another 20% of trials, the first lick triggered optogenetic stimulation, which lasted until 1.5s after stimulus onset. We obtained similar, albeit weaker effects compared to the trials with earlier optogenetic stimulation (maximally 0.5 licks difference between no stimulation and stimulation conditions). Given that lick onset times were also variable, we focused our main analysis on trials in which optogenetic stimulation started 0.5s prior to the onset of the visual stimulus.

For behavioral analysis related to Figure 6A, we included genotype (D1 and D2), optogenetic stimulation epoch, and visual stimulus side as factors in the mixed-effects model, predicting either accuracy (Equation 2) or Omission (Equation 3). Post-hoc Wald tests of coefficients were then performed per genotype, and per stimulation epoch, to determine the difference in accuracy between the two stimulus sides. Post-hoc tests were corrected for multiple comparisons.

Mixed-effect models were also applied to neural data (i.e., predicting ΔF/F). This was done separately per brain region, timepoint, and genotype.

#### Decoding strategies

We applied multi-pixel pattern decoding strategies to determine how well brain activity could be used to decode the side of the visual stimulus and the lick response (Figure S4). Decoding accuracy was calculated by comparing the predicted label to the actual labels. To prevent biasing the decoding models, we selected an equal number of trials from each condition, 25% of correct and 25% error trials with stimulus_ContraOpto_ and 25% of correct and 25% error trials with stimulus_IpsiOpto_. We used 5-fold cross-validation, training the model on 80 percent of data to predict the labels of the other 20 percent. We used the “least dirty” method from the multi-task learning for structural regularization (MALSAR) toolbox for MATLAB.^57^ MALSAR assigned weights to as few pixels as possible to decode the side of the stimulus and the lick response. We ran the model on data from different epochs in a trial, for individual mice. In some instances, we trained the model on data from a certain condition (e.g., optogenetic stimulation OFF) and tested the model on data from another condition (e.g., early optogenetic stimulation), e.g., in the analysis of Figure 6D. To establish significance of the predictions and beta-values for individual mice, we performed bootstrapping (i.e., repeated the procedure 1,000 times while randomizing trial labels with replacement). The significance was determined as the percentile of the prediction in the distribution obtained by bootstrapping.

#### Linear models to understand the nature of variability in cortical activity

Changes in cortical activity can be a consequence of many factors, which we disentangled with linear models. We use kernel ridge regression similar as described by.^55^

The goal of the model is to create an estimate $\hat{y}$ of *y*, which is ΔF/F across time for each pixel, such that the term $\sum {\left(\hat{y}-y\right)}^{2}$ is minimized. This difference can be expressed as fraction of the total variance explained by the model (Figures S3A and S3B):

(Equation 6) $$R^{2}=1-\frac{\sum {(y_{test}-\hat{y)}}^{2}}{\sum {(y_{test}-\bar{y_{test})}}^{2}}$$

in which

(Equation 7) $$\hat{y}=X_{test}\beta$$

We used 3-fold cross-validation such that a third of all trials were used as *test* in every fold, and the rest as *train* (randomly assigned). X is a ‘Design Matrix’ with columns that represent the side of the visual stimulus, optogenetic stimulation, left and right lick responses, body movement, eye movements, and the intercept, all normalized between 0 and 1. The values of β define the model and are estimated as follows:

(Equation 8) $$\beta ={\left({X_{train}}^{T}X_{train}+\alpha I\right)}^{-1}{X_{train}}^{T}y_{train},with\;\alpha \left\{0:0.5:50\right\}$$

α was chosen such that $\sum {\left(\hat{y}-y\right)}^{2}$ was minimized for each pixel independently in a nested-cross validation (i.e., one-third of the training data was left out within the main fold, to define the best α in the training set), and *I* is the identity matrix.

In kernel regression, the task-parameters are replicated across timepoints (i.e., we repeated each task-event across all time points of a trial, see Figure S3A), allowing the study of the activity dynamics. Continuous predictors (i.e., body- and eye-movements) were added as normalized values at the timepoint of occurrence. To evaluate which parameters contributed significantly to the total variance explained, we repeated the above analysis while circularly shifting the parameter of interest. For example, we shifted the columns of X representing the visual stimulus 500 times, while keeping the structure in replications across timepoints (Figure S3B). As a measure of minimum unique contribution of the shifted parameter to the model we calculated Δ $R^{2}$.

(Equation 9) $$\Delta R^{2}=R^{2}\;-\;R_{shift}^{2}$$

The effect of e.g., visual stimulus presentation was considered to be significant if the total variance explained by the original model ($R^{2})$ was above the 95^th^ percentile of the distribution of circularly shifted variance explained ($R_{shift}^{2})$ (Figure S3D). For the left hemisphere of two mice, $R^{2}$ was not above the 95^th^ percentile for any of the parameters of interest (probably due to the optic fiber implant), hence these data were excluded from further statistical analysis.

## References

[1] Fuster J.M., Alexander G.E. (1973). Firing changes in cells of the nucleus medialis dorsalis associated with delayed response behavior. Brain Res..

[2] Kubota K., Niki H. (1971). Prefrontal cortical unit activity and delayed alternation performance in monkeys. J. Neurophysiol..

[3] Fuster J.M., Jervey J.P. (1981). Inferotemporal neurons distinguish and retain behaviorally relevant features of visual stimuli. Science.

[4] Curtis C.E., D’Esposito M. (2003). Persistent activity in the prefrontal cortex during working memory. Trends Cognit. Sci..

[5] Van Kerkoerle T., Self M.W., Roelfsema P.R. (2017). Layer-specificity in the effects of attention and working memory on activity in primary visual cortex. Nat. Commun..

[6] Mendoza-Halliday D., Torres S., Martinez-Trujillo J.C. (2014). Sharp emergence of feature-selective sustained activity along the dorsal visual pathway. Nat. Neurosci..

[7] van Vugt B., van Kerkoerle T., Vartak D., Roelfsema P.R. (2020). The contribution of AMPA and NMDA receptors to persistent firing in the dorsolateral prefrontal cortex in working memory. J. Neurosci..

[8] Fuster J.M., Alexander G.E. (1971). Neuron activity related to short-term memory. Science.

[9] Goard M.J., Pho G.N., Woodson J., Sur M. (2016). Distinct roles of visual, parietal, and frontal motor cortices in memory-guided sensorimotor decisions. Elife.

[10] Guo Z.V., Inagaki H.K., Daie K., Druckmann S., Gerfen C.R., Svoboda K. (2017). Maintenance of persistent activity in a frontal thalamocortical loop. Nature.

[11] Christophel T.B., Klink P.C., Spitzer B., Roelfsema P.R., Haynes J.-D. (2017). The Distributed Nature of Working Memory. Trends Cognit. Sci..

[12] Rainer G., Rao S.C., Miller E.K. (1999). Prospective coding for objects in primate prefrontal cortex. J. Neurosci..

[13] Peixoto D., Verhein J.R., Kiani R., Kao J.C., Nuyujukian P., Chandrasekaran C., Brown J., Fong S., Ryu S.I., Shenoy K.V., Newsome W.T. (2021). Decoding and perturbing decision states in real time. Nature.

[14] Ding L., Gold J.I. (2010). Caudate encodes multiple computations for perceptual decisions. J. Neurosci..

[15] D’Esposito M., Postle B.R. (2015). The Cognitive Neuroscience of Working Memory. Annu. Rev. Psychol..

[16] Baddeley A. (1992). Working memory. Science.

[17] Dotson N.M., Hoffman S.J., Goodell B., Gray C.M. (2018). Feature-Based Visual Short-Term Memory Is Widely Distributed and Hierarchically Organized. Neuron.

[18] Voitov I., Mrsic-Flogel T.D. (2022). Cortical feedback loops bind distributed representations of working memory. Nature.

[19] Chen T.W., Li N., Daie K., Svoboda K. (2017). A Map of Anticipatory Activity in Mouse Motor Cortex. Neuron.

[20] Esmaeili V., Tamura K., Muscinelli S.P., Modirshanechi A., Boscaglia M., Lee A.B., Oryshchuk A., Foustoukos G., Liu Y., Crochet S. (2021). Rapid suppression and sustained activation of distinct cortical regions for a delayed sensory-triggered motor response. Neuron.

[21] Wang L., Rangarajan K.V., Gerfen C.R., Krauzlis R.J. (2018). Activation of Striatal Neurons Causes a Perceptual Decision Bias during Visual Change Detection in Mice. Neuron.

[22] Wang Y., Yin X., Zhang Z., Li J., Zhao W., Guo Z.V. (2021). A cortico-basal ganglia-thalamo-cortical channel underlying short-term memory. Neuron.

[23] Wang L., Krauzlis R.J. (2020). Involvement of Striatal Direct Pathway in Visual Spatial Attention in Mice. Curr. Biol..

[24] Lee J., Wang W., Sabatini B.L. (2020). Anatomically segregated basal ganglia pathways allow parallel behavioral modulation. Nat. Neurosci..

[25] Oh S.W., Harris J.A., Ng L., Winslow B., Cain N., Mihalas S., Wang Q., Lau C., Kuan L., Henry A.M. (2014). A mesoscale connectome of the mouse brain. Nature.

[26] Haber S.N. (2016). Corticostriatal circuitry. Dialogues Clin. Neurosci..

[27] McGeorge a J., Faull R.L. (1989). The organization of the projection from the cerebral cortex to the striatum in the rat. Neuroscience.

[28] Saint-Cyr J.A., Ungerleider L.G., Desimone R. (1990). Organization of visual cortical inputs to the striatum and subsequent outputs to the pallido-nigral complex in the monkey. J. Comp. Neurol..

[29] Alexander G.E., DeLong M.R., Strick P.L. (1986). Parallel organization of functionally segregated circuits linking basal ganglia and cortex. Annu. Rev. Neurosci..

[30] Foster N.N., Barry J., Korobkova L., Garcia L., Gao L., Becerra M., Sherafat Y., Peng B., Li X., Choi J.-H. (2021). The mouse cortico–basal ganglia–thalamic network. Nature.

[31] Peters A.J., Fabre J.M.J., Steinmetz N.A., Harris K.D., Carandini M. (2021). Striatal activity reflects cortical activity patterns. Nature.

[32] Choi E.Y., Yeo B.T.T., Buckner R.L. (2012). The organization of the human striatum estimated by intrinsic functional connectivity. J. Neurophysiol..

[33] Wang X.J. (2001). Synaptic reverberation underlying mnemonic persistent activity. Trends Neurosci..

[34] Saunders A., Oldenburg I.A., Berezovskii V.K., Johnson C.A., Kingery N.D., Elliott H.L., Xie T., Gerfen C.R., Sabatini B.L. (2015). A direct GABAergic output from the basal ganglia to frontal cortex. Nature.

[35] Wilhelm M., Sych Y., Fomins A., Alatorre Warren J.L., Lewis C., Serratosa Capdevila L., Boehringer R., Amadei E.A., Grewe B., O’Connor E.C. (2023). Striatum-projecting prefrontal cortex neurons support working memory maintenance. Nat. Commun..

[36] Hikosaka O., Wurtz R.H. (1983). Visual and oculomotor functions of monkey substantia nigra pars reticulata. I. Relation of visual and auditory responses to saccades. J. Neurophysiol..

[37] Hikosaka O., Sakamoto M., Usui S. (1989). Functional properties of monkey caudate neurons. III. Activities related to expectation of target and reward. J. Neurophysiol..

[38] Mushiake H., Strick P.L. (1995). Pallidal neuron activity during sequential arm movements. J. Neurophysiol..

[39] Gerfen C.R., Surmeier D.J. (2011). Modulation of striatal projection systems by dopamine. Annu. Rev. Neurosci..

[40] Nonomura S., Nishizawa K., Sakai Y., Kawaguchi Y., Kato S., Uchigashima M., Watanabe M., Yamanaka K., Enomoto K., Chiken S. (2018). Monitoring and Updating of Action Selection for Goal-Directed Behavior through the Striatal Direct and Indirect Pathways. Neuron.

[41] Tecuapetla F., Jin X., Lima S.Q., Costa R.M. (2016). Complementary Contributions of Striatal Projection Pathways to Action Initiation and Execution. Cell.

[42] Smith Y., Bevan M.D., Shink E., Bolam J.P. (1998). Microcircuitry of the direct and indirect pathways of the basal ganglia. Neuroscience.

[43] Lee J., Sabatini B.L. (2021). Striatal indirect pathway mediates exploration via collicular competition. Nature.

[44] Cruz B.F., Guiomar G., Soares S., Motiwala A., Machens C.K., Paton J.J. (2022). Action suppression reveals opponent parallel control via striatal circuits. Nature.

[45] Cox J., Witten I.B. (2019). Striatal circuits for reward learning and decision-making. Nat. Rev. Neurosci..

[46] Schultz W., Dayan P., Montague P.R. (1997). A neural substrate of prediction and reward. Science.

[47] Schultz W. (2016). Dopamine reward prediction- error signalling: a two-component response. Nat. Rev. Neurosci..

[48] Znamenskiy P., Zador A.M. (2013). Corticostriatal neurons in auditory cortex drive decisions during auditory discrimination. Nature.

[49] Xiong Q., Znamenskiy P., Zador A.M. (2015). Selective corticostriatal plasticity during acquisition of an auditory discrimination task. Nature.

[50] Frank M.J., Loughry B., O’Reilly R.C. (2001). Interactions between frontal cortex and basal ganglia in working memory: a computational model. Cognit. Affect Behav. Neurosci..

[51] Wise S.P., Murray E.A., Gerfen C.R. (1996). The frontal cortex-basal ganglia system in primates. Crit. Rev. Neurobiol..

[52] Dana H., Chen T.-W., Hu A., Shields B.C., Guo C., Looger L.L., Kim D.S., Svoboda K. (2014). Thy1-GCaMP6 Transgenic Mice for Neuronal Population Imaging In Vivo. PLoS One.

[53] Ren C., Komiyama T. (2021). Characterizing cortex-wide dynamics with wide-field calcium imaging. J. Neurosci..

[54] Guo Z.V., Li N., Huber D., Ophir E., Gutnisky D., Ting J.T., Feng G., Svoboda K. (2014). Flow of cortical activity underlying a tactile decision in mice. Neuron.

[55] Musall S., Kaufman M.T., Juavinett A.L., Gluf S., Churchland A.K. (2019). Single-trial neural dynamics are dominated by richly varied movements. Nat. Neurosci..

[56] Orsolic I., Rio M., Mrsic-Flogel T.D., Znamenskiy P. (2021). Mesoscale cortical dynamics reflect the interaction of sensory evidence and temporal expectation during perceptual decision-making. Neuron.

[57] Zhou J., Chen J., Ye J. (2012). MALSAR: Multi-Task Learning via Structural Regularization. http://www.MALSAR.org.

[58] Groenewegen H.J. (2003). The basal ganglia and motor control. Neural Plast..

[59] Graybiel A.M. (1998). The Basal Ganglia and Chunking of Action Repertoirs. Neurobiol. Learn. Mem..

[60] White N.M. (1997). Mnemonic functions of the basal ganglia. Curr. Opin. Neurobiol..

[61] Lee J.Y., Jun H., Soma S., Nakazono T., Shiraiwa K., Dasgupta A., Nakagawa T., Xie J.L., Chavez J., Romo R. (2021). Dopamine facilitates associative memory encoding in the entorhinal cortex. Nature.

[62] Bolkan S.S., Stone I.R., Pinto L., Ashwood Z.C., Iravedra Garcia J.M., Herman A.L., Singh P., Bandi A., Cox J., Zimmerman C.A. (2022). Opponent control of behavior by dorsomedial striatal pathways depends on task demands and internal state. Nat. Neurosci..

[63] Rossi M.A., Li H.E., Lu D., Kim I.H., Bartholomew R.A., Gaidis E., Barter J.W., Kim N., Cai M.T., Soderling S.H. (2016). A GABAergic nigrotectal pathway for coordination of drinking behavior. Nat. Neurosci..

[64] Oldenburg I.A., Sabatini B.L. (2015). Antagonistic but Not Symmetric Regulation of Primary Motor Cortex by Basal Ganglia Direct and Indirect Pathways. Neuron.

[65] Tai L.H., Lee A.M., Benavidez N., Bonci A., Wilbrecht L. (2012). Transient stimulation of distinct subpopulations of striatal neurons mimics changes in action value. Nat. Neurosci..

[66] Lee H.J., Weitz A.J., Bernal-casas D., Duffy B.A., Choy M., Kravitz A.V., Kreitzer A.C., Lee J.H. (2016). Activation of Direct and Indirect Pathway Medium Spiny Neurons Drives Distinct Brain-wide Responses. Neuron.

[67] Sippy T., Lapray D., Crochet S., Petersen C.C.H. (2015). Cell-Type-Specific Sensorimotor Processing in Striatal Projection Neurons during Goal-Directed Behavior. Neuron.

[68] Chun M.M., Turk-Browne N.B. (2007). Interactions between attention and memory. Curr. Opin. Neurobiol..

[69] Reeves A., Sperling G. (1986). Attention Gating in Short-Term Visual Memory. Psychol. Rev..

[70] Gao Z., Davis C., Thomas A.M., Economo M.N., Abrego A.M., Svoboda K., De Zeeuw C.I., Li N. (2018). A cortico-cerebellar loop for motor planning. Nature.

[71] Schnabel U.H., Van der Bijl T., Roelfsema P.R., Lorteije J.A.M. (2021). A direct comparison of spatial attention and stimulus–response compatibility between mice and humans. J. Cognit. Neurosci..

[72] Alkire M.T., Hudetz A.G., Tononi G. (2008). Consciousness and Anesthesia. Science.

[73] Klapoetke N.C., Murata Y., Kim S.S., Pulver S.R., Birdsey-Benson A., Cho Y.K., Morimoto T.K., Chuong A.S., Carpenter E.J., Tian Z. (2014). Independent optical excitation of distinct neural populations. Nat. Methods.

[74] Stujenske J.M., Spellman T., Gordon J.A. (2015). Modeling the Spatiotemporal Dynamics of Light and Heat Propagation for InVivo Optogenetics. Cell Rep..

[75] Hartman J. (2019). https://github.com/jackatta/estimated-marginal-means.

[76] Marshel J.H., Garrett M.E., Nauhaus I., Callaway E.M. (2011). Functional specialization of seven mouse visual cortical areas. Neuron.

[77] Guo Z.V., Hires S.A., Li N., O’Connor D.H., Komiyama T., Ophir E., Huber D., Bonardi C., Morandell K., Gutnisky D. (2014). Procedures for Behavioral Experiments in Head-Fixed Mice. PLoS One.

[78] Owen S.F., Liu M.H., Kreitzer A.C. (2019). Thermal constraints on in vivo optogenetic manipulations. Nat. Neurosci..

[79] van Beest E.H., Mukherjee S., Kirchberger L., Schnabel U.H., van der Togt C., Teeuwen R.R.M., Barsegyan A., Meyer A.F., Poort J., Roelfsema P.R., Self M.W. (2021). Mouse visual cortex contains a region of enhanced spatial resolution. Nat. Commun..

[80] Allen Institute for Brain Science (2017).

