# Supplementary material for: The direct and indirect pathways of the basal ganglia antagonistically influence cortical activity and perceptual decisions
Source: iScience. 2024 Aug 22;27(9):110753. doi: 10.1016/j.isci.2024.110753 (PMC11402218; doi:10.1016/j.isci.2024.110753)
Supplement: Document S1. Figures S1–S6 and Tables S1 and S2 [file mmc1.pdf]

iScience, Volume *27*

Supplemental information

The direct and indirect pathways of the basal ganglia antagonistically influence cortical activity and perceptual decisions

Enny H. van Beest, Mohammed A.O. Abdelwahab, J. Leonie Cazemier, Chrysiida Baltira, M. Cassandra Maes, Brandon D. Peri, Matthew W. Self, Ingo Willuhn, and Pieter R. Roelfsema

Supplementary Figures

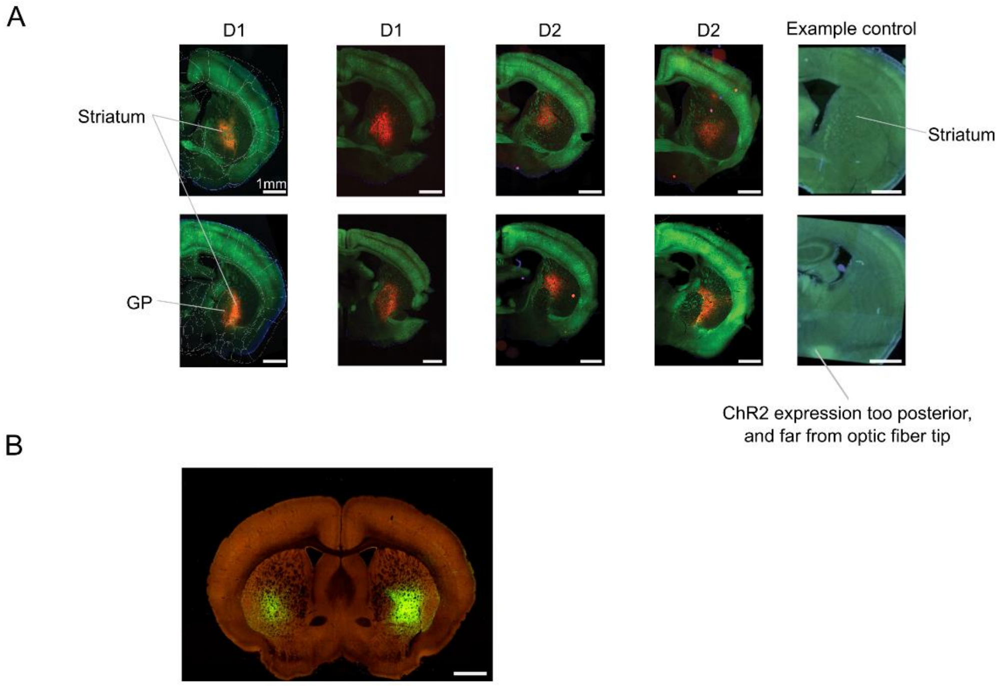

Figure S1: Histology details, related to Figure 1. A) Expression of Thy1-GCaMP-GFP (green) and ChrimsonR-tdTomato (red) in example D1-cre/D2-cre X Thy1-GCaMP6f mice and one control mouse in which no ChR2 was expressed in this regions of the striatum. Top row is approximately +0.14mm anterior to Bregma, and bottom row 0.4mm posterior to Bregma. ChrimsonR-tdTomato positive cell bodies were mainly found in the striatum. Scale bars indicate 1mm on all images. B) The Mouse Connectivity atlas of Allen Brain Institute reveals that the same region is connected to the secondary motor cortex (ALM region; image 51 of experiment 157710335 of the atlas). Scale bar indicates 1mm.

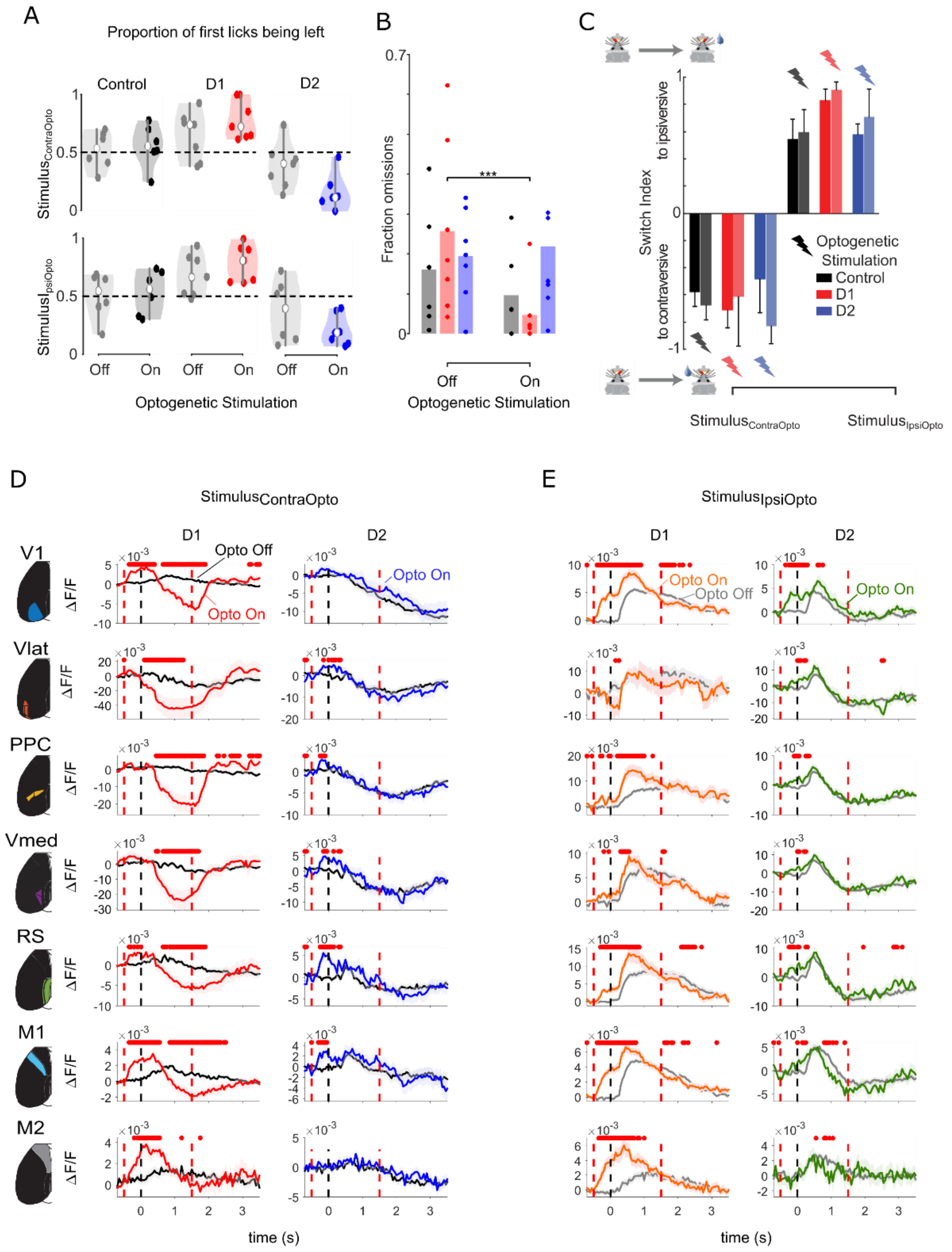

Figure S2: Effect of optogenetic stimulation of the striatum in a visual detection task, related to Figure 4. A) Proportion of first licks after stimulus onset that are contralateral to the stimulation site on trials with optogenetic stimulation (black/red/blue) or without (grey) for individual mice. Top (bottom) row: $\text{stimulus}_{\text{ContraOpto}}$ ($\text{stimulus}_{\text{IpsiOpto}}$). A mixed effects model with factors genotype, stimulus side and optogenetic stimulation (mouse as random factor) revealed a significant interaction ($F_{2,68} = 11.6$, $p<0.001$). Specifically, D2 mice had a lower proportion of contraversive first licks than D1 and control mice ($p<0.001$). The main effect of optogenetic stimulation was significant for D1 ($p<0.05$) and D2 ($p<0.001$) mice. There were no significant differences between the genotypes in trials without optogenetic stimulation (ps>0.35). B) Influence of optogenetic stimulation on the fraction of omissions. Optogenetic stimulation in D1-cre mice decreased the number of omissions. ***, $p<0.001$ for post-hoc Wald test of coefficients. C) Average switch index (SI), which is negative if mice tend to switch to contraversive licks (i.e. when reward is delivered via the lick spout opposite to the optogenetic stimulation side) and positive otherwise. As expected, the

location of the visual stimulus (and therefore reward) influenced the switch index (repeated measures ANOVA, $F_{1,65}=231$, $p<0.001$), but optogenetic stimulation did not. D) Time-courses of GCaMP signal in ROIs of $\text{hemisphere}_{\text{Control}}$ in D1-cre (N=4) and D2-cre mice (N=3 mice) (same as Figure 4E, but now in $\text{hemisphere}_{\text{Control}}$) for $\text{stimulus}_{\text{ContraOpto}}$ trials. Red/blue curves for optogenetic stimulation on, black for optogenetic stimulation off. Vertical dashed lines indicate the boundaries between the epochs: pre-stimulus optogenetic stimulation onset (red), visual stimulus onset (black), and post-trial onset (red). Shaded area denotes s.e.m. The red dots above indicate a significant main effect of optogenetic stimulation ($p<0.05$) Note that part of $\text{hemisphere}_{\text{Control}}$ was occluded by the fiber implant. E) same as in C, but now for $\text{stimulus}_{\text{IpsiOpto}}$ trials. Orange/green curves for optogenetic stimulation on, gray for optogenetic stimulation off.

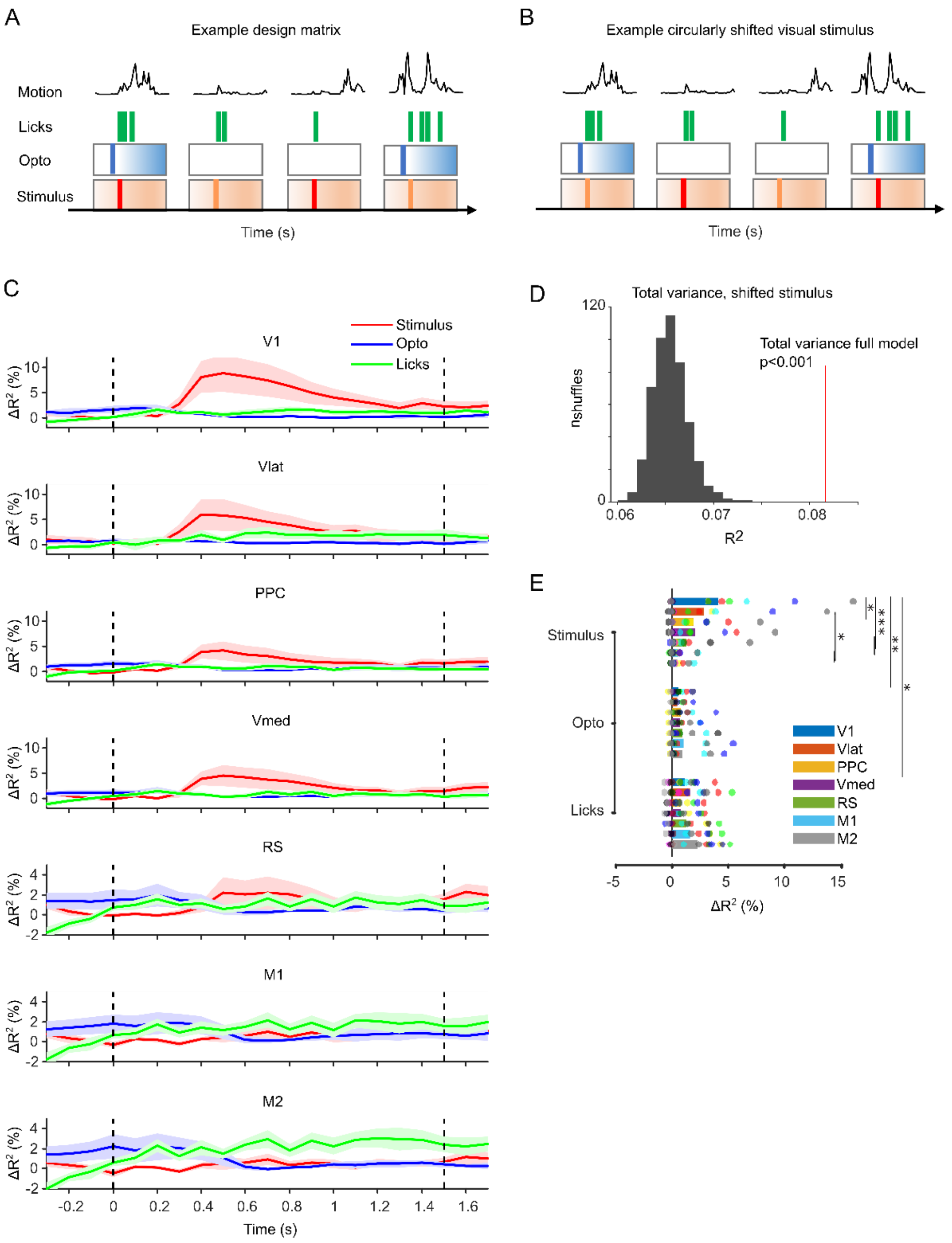

Figure S3: Linear models for the visual detection task, related to Figure 4. A) Schematic of design matrix with kernel regression. The timing of predictors during four trials are illustrated: stimulus onsets (red and orange lines for contra and ipsilateral stimuli), optogenetic stimulation onsets (blue lines), licks (green lines) and a continuous motion signal. To account for dynamics in activity we included regressors for the visual and optogenetic stimulus at every time point (red and blue gradient). B) Example design matrix with circularly shifted visual stimulus (orange and red lines are shifted). C) Average +/- s.e.m. unique explained variance (ΔR^2) over time for parameters in a linear model: visual stimulus (red), optogenetic stimulation (blue), and licks (green). Vertical dashed lines indicate visual stimulus onset and optogenetic stimulation offset. Rows for different ROIs. D) Distribution of the variance explained (R^2_{shift}) after circularly shifting the visual stimulus predictor in the design matrix 500 times (i.e. shifting by entire trials). Red line, variance explained by the full model (R^2). In this example R^2 was larger than all R^2_{shift} values. E) Average +/- s.e.m. ΔR^2 (%) bar plots for different ROIs (different bar colors as indicated) and variables (visual stimulus, optogenetic stimulation, licks) for the visual + optogenetic stimulation epoch (0-1.5s). Mice are shown with different colors (each mouse contributes two data points, one per hemispheres). Post hoc t-tests (Bonferroni corrected): *, p<0.05; **, p<0.01; ***, p<0.001.

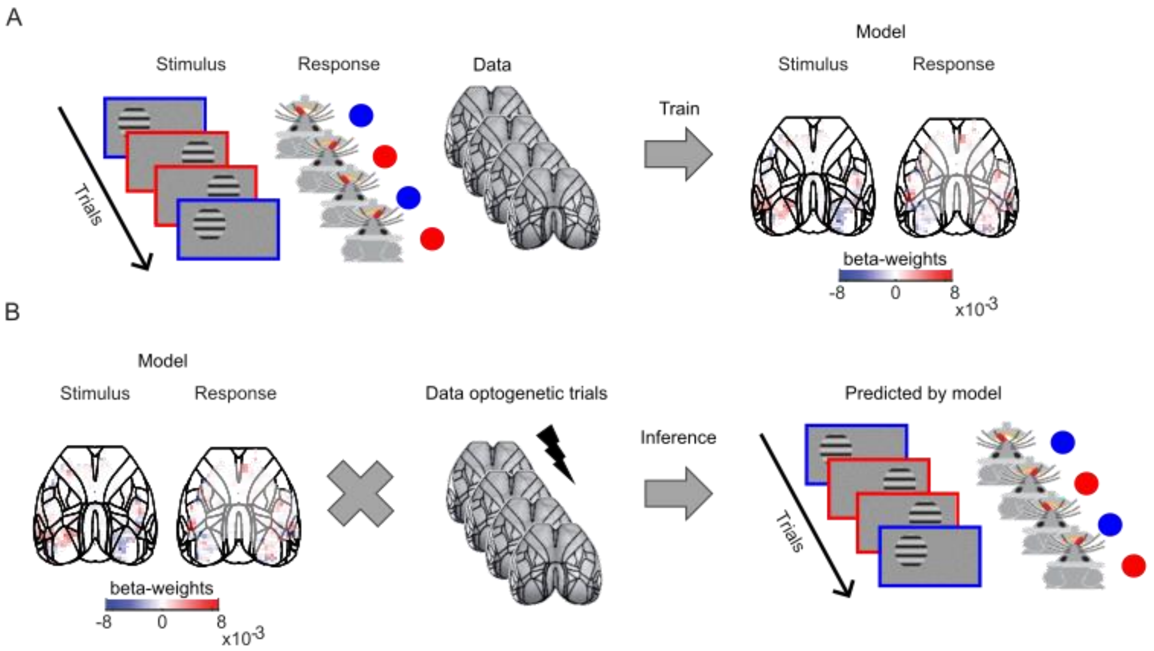

Figure S4: Multi output decoding with MALSAR, related to Figures 5 and 6. A) We trained a model using the "least dirty" method from the multi-task learning for structural regularization (MALSAR) toolbox for MATLAB[57]. This method assigns weights to as few pixels as possible, to simultaneously decode the side of the stimulus and the lick response. It takes the shared and unique activity patterns related to the stimulus and the lick response into account. The left (right) stimulus is indicated by a blue (red) rectangle and a leftward (rightward) lick with a blue (red) circle. The mice make errors, i.e. they lick leftward for a stimulus on the right and vice versa. When training the model we balanced the trials to get an equal amount of all trial types (left stimulus left response, left stimulus right response, right stimulus right response, right stimulus left response). B) We inferred the predicted stimulus and lick response on trials held out during model construction, e.g. from trials with optogenetic stimulation.

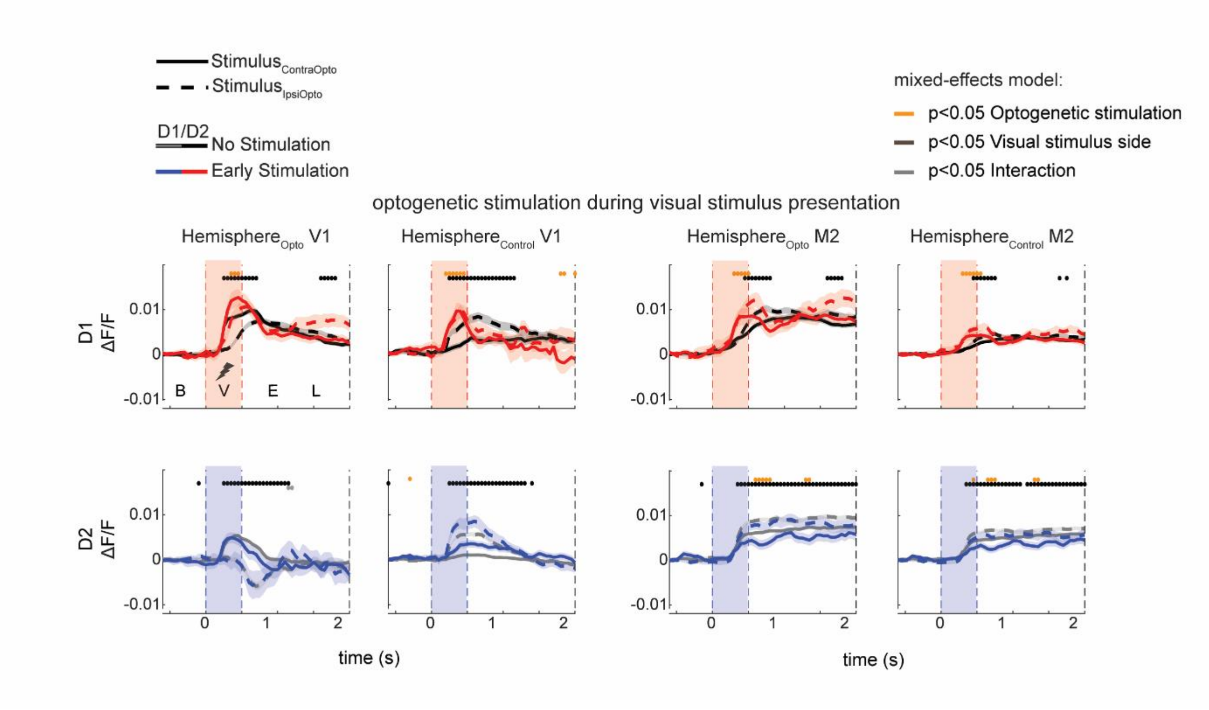

Figure S5: Optogenetic stimulation of the striatum in the delayed response task, related to Figure 6. Influence of optogenetic stimulation of the striatum (colored traces) during the presentation of the visual stimulus (0-0.5s after stimulus onset) on activity in M2 and V1. Traces show average $\Delta F/F$ and shaded regions s.e.m. Solid (dashed) lines represent trials with stimulus$_{ContraOpto}$ (stimulus$_{IpsiOpto}$). Mixed-effects models per time point revealed significant main effects of optogenetic stimulation (orange circles, $p<0.05$), visual stimulus side (black circles, $p<0.05$) and the interaction between these factors (grey circles, $p<0.05$).

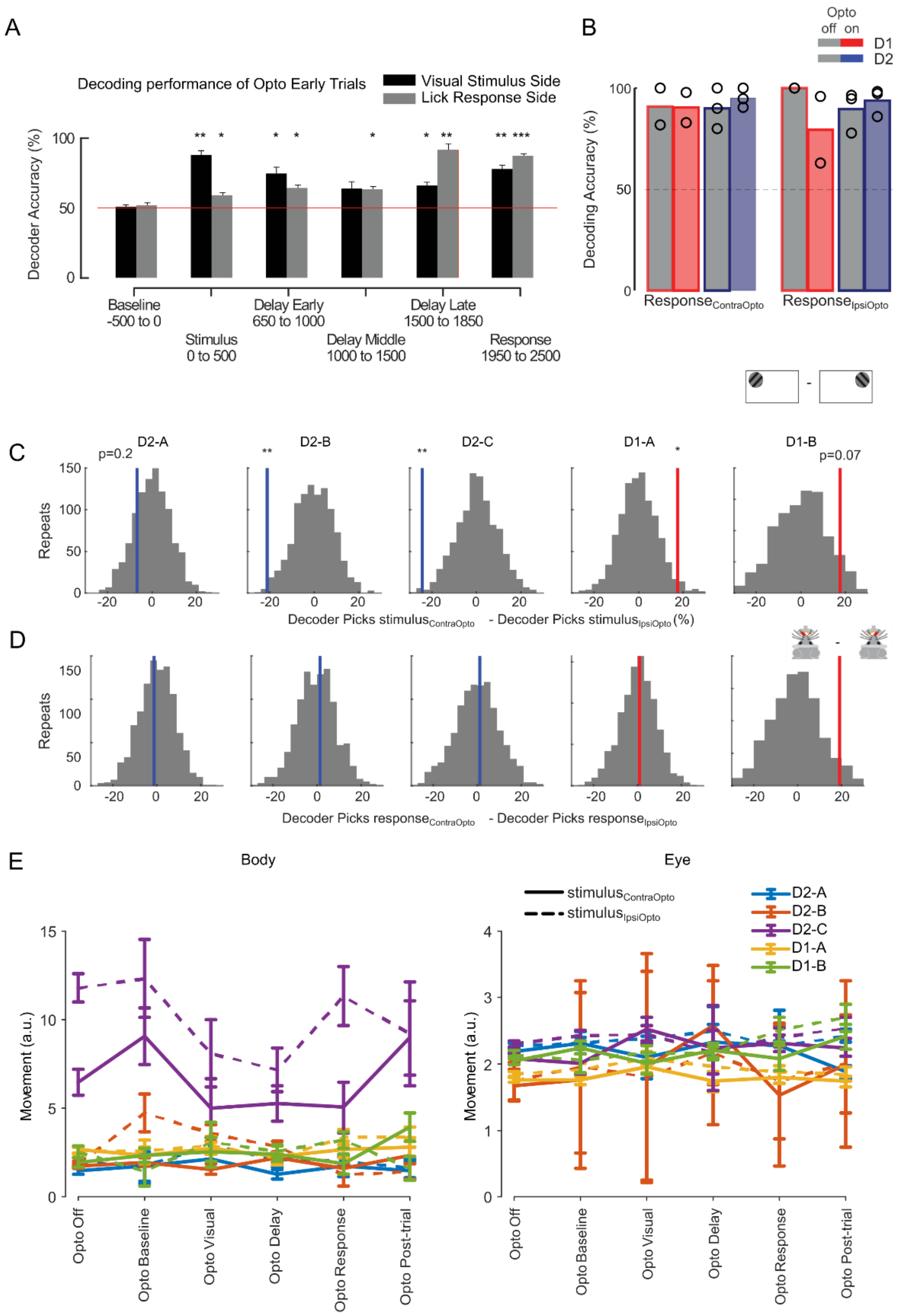

Figure S6: MALSAR decoding of visual stimulus and lick direction and the influence of optogenetic activation, related to Figure 6. A) Decoding accuracy of stimulus location (black bars) and lick direction (grey bars) in D-creXThy1-GCaMp6f mice in trials with early optogenetic stimulation using a model that was trained on trials without optogenetic stimulation. Stars indicate significance; *, $p<0.05$; **, $p<0.01$; ***, $p<0.001$. B) same as **Figure6D**, but for response decoding. There were no significant effects. C) Blue and red lines show the influence of optogenetic stimulation on stimulus decoding in individual D1-cre and D2-cre mice. Positive (negative) values indicate an increase in the decoding of stimulus$_{ContraOpto}$ (stimulus$_{IpsiOpto}$). Histograms show the bootstrapped distributions. *, $p<0.05$; **, $p<0.01$. D) Same as in C, but for response$_{ContraOpto}$ (response$_{IpsiOpto}$). E) Median +/- s.e.m. (across trials) movement (a.u.) per mouse in the late delay time window, for different optogenetic stimulation conditions (x-axis). Solid (dashed) lines are trials with stimulus$_{ContraOpto}$ (stimulus$_{IpsiOpto}$). Left, Body movements measured by a piezo element under the front paws of the mouse. Right: Eye movements, measured as the average difference in z-scored x-position, y-position, width or height of the pupil between subsequent timepoints.

Acronym	Full name	Part of
**V1**	Primary visual cortex	Dorsal cortex
**Vlat**	Lateral visual cortex	Dorsal cortex
**PPC**	posterior parietal cortex	Dorsal cortex
**Vmed**	medial visual cortex	Dorsal cortex
**RS**	Retrosplenial cortex	Dorsal cortex
**M1**	Primary motor cortex	Dorsal cortex
**M2**	Secondary motor cortex	Dorsal cortex
**D1**	Dopamine-1 (Receptor)	Direct pathway basal ganglia
**D2**	Dopamine-2 (Receptor)	Indirect pathway basal ganglia
**dSPN**	Direct-pathway striatal projection neurons	Basal ganglia (direct pathway)
**iSPN**	Indirect-pathway striatal projection neurons	Basal ganglia (indirect pathway)

Table S1: Acronyms, related to Figures 1 and 2, and STAR Methods. Brain regions and receptors, their full name and whether they belong to cortex or basal ganglia.

ID	Genotype & Virus	Sex (M/F)	Fig. 1e	Fig. 2	Fig. 3-4; S2A-B behavior	Fig. 3-4; S2C neural	Fig. S4	Fig. 5	Fig. 6; S6
**1**	D2XChrimsonRXGCaMP	F	X	X		X		X	X
**2**	D1XChrimsonRXGCaMP	F	X	X	X	X		X	X
**3**	D2XChrimsonRXGCaMP	F	X	X	X	X		X	X
**4**	D1XChrimsonRXGCaMP	M	X	X	X	X	X		
**5**	D2XChrimsonRXGCaMP	M	X	X	X	X		X	X
**6**	D1XChrimsonRXGCaMP	F		X	X	X			
**7**	D1XChrimsonRXGCaMP	F	X	X	X	X	X	X	X
**8**	D1XChrimsonRXGCaMP	F	X	X					
**9**	D2XChr2	M	X		X				
**10**	D2XChrimsonR	F	X		X				
**11**	D1XChr2	M	X		X				
**12**	D1XChr2	M	X		X				
**13**	D2XChrimsonRXGCaMP	M	X						
**14**	D2XChr2 (control)	M			X				
**15**	D1XChr2 (control)	M			X				
**16**	D1XChr2 (control)	M			X				
**17**	D1XChr2	M	X		X				
**18**	D2XChr2	F	X		X				
**19**	D2XChrimsonR (control)	F			X				
**20**	D1XChrimsonR (control)	F			X				
**21**	D2XChr2 (control)	M			X				
**22**	D2XChr2	M	X		X				
**23**	D2XChr2	M	X		X				
**24**	GCaMP	M						X	
**25**	GCaMP	M						X	
**26**	GCaMP	F						X	
**27**	GCaMP	F						X	
**28**	GCaMP	M						X	

Table S2: Mice that contributed to the main results, related to Figures 1-6, and STAR Methods. D1/D2 refers to D1 or D2 – cre positive mice, ChrimsonR and Chr2 are both DIO-versions, such that the excitatory opsin is only expressed in D1 or D2 cells. GCaMP stands for Thy1-GCaMP6f positive mice. A cell in the table is marked with an X if a mouse contributed to the data in a particular figure. X in bold is the example mouse (if applicable). Mice that were excluded from analysis are not shown.
